# 1 km HILDA + based land cover/use map time series of China under 1.5 °C climate of this century

**DOI:** 10.1038/s41597-025-06411-9

**Published:** 2025-12-11

**Authors:** Yifan Gao, Xian Feng, Changqing Song, Yuanhui Wang, Sijing Ye, Min Zhao, Delin Fang, Peichao Gao

**Affiliations:** State Key Laboratory of Earth Surface Processes and Disaster Risk Reduction, Faculty of Geographical Science, Beijing Normal University, Beijing, 100875 China

**Keywords:** Climate-change mitigation, Climate and Earth system modelling

## Abstract

Limiting the global temperature rise in this century to 1.5 °C above preindustrial levels has become a critical challenge. One key pathway involves actions in the land sector. Forecasts of land cover/use maps can provide valuable insights for guiding such actions, but the reliability of maps strongly depends on the accuracy of the baseline land cover/use map. In this study, we selected an accurate land cover/use map—HIstoric Land Dynamics Assessment+ (HILDA+), harmonized with multiple historical land cover/use maps—as the baseline land cover/use map. Additionally, we selected a future scenario that aims to achieve the 1.5 °C target and takes into account nationally determined contributions (NDCs). This scenario assumes that all parties would adhere to their submitted NDCs. We forecasted land cover/use maps at a spatial resolution of 1 km from 2020 to 2100 at a 10-year interval in China by integrating the Global Change Analysis Model, the Land-N2N model, and HILDA + land cover/use maps. These maps provide scientific guidance to support land management in addressing climate crises.

## Background & Summary

A rapid increase in temperature has raised widespread concern^1,2^. A targeted effort to limit the global average temperature rise to 1.5 °C above preindustrial levels was proposed in the Paris Agreement^3^. In the 26th Conference of Parties (COP26) in Glasgow, 154 parties submitted or updated their nationally determined contributions (NDCs) to limit the rapid increase in temperature and address the climate crisis^3,4^. As of March 2025, a total of 213 parties have submitted or updated their NDCs (https://unfccc.int/NDCREG). Additionally, the United Nations launched the Climate Promise 2025 initiative (https://climatepromise.undp.org/what-we-do/flagship-initiatives/climate-promise-2025) to support countries in aligning their NDCs with the 1.5 °C target.

Taking action on land cover/use change is essential for limiting the rapid temperature increase^5,6^. The rapid increase in temperature is largely due to the accumulation of carbon in the atmosphere. Among the various contributors to carbon emissions, land cover/use change should not be ignored^7–10^. According to existing research, carbon emissions resulting from land cover/use change account for approximately one-third of all anthropogenic carbon emissions^11^. Specifically, deforestation contributes significantly to carbon emissions^7,12^. Therefore, land management is prominent in NDCs. For example, the updated NDC of New Zealand in 2025 reported that “Appropriate incentives will be required to balance encouraging afforestation for increased sequestration with other land uses”. The updated NDC of Canada in 2025 reported that funds would be “Invested in reforestation, fertilization, tree improvement, and road rehabilitation”.

Multiple forecasts of land cover/use maps have been published to support research and assist decision-makers in developing land management policies. Most existing forecasts of land cover/use maps focus on land changes under shared socioeconomic pathways (SSPs) and representative concentration pathways (RCPs)^13,14^. SSPs represent a range of potential future social and economic possibilities^15,16^. RCPs represent future climate change possibilities based on different levels of radiative forcing^15^. For example, Luo *et al*.^13^ forecasted land changes in China from 2020 to 2100 under both SSPs and RCPs. Only a few forecasts of land cover/use maps take into account NDCs^17–19^. For example, Gao *et al*.^19^ forecasted global land system changes under three scenarios from 2015 to 2100 at a 5 km resolution. Compared with forecasts of land cover/use maps under SSPs or RCPs, forecasts of land cover/use maps that account for NDCs are more suitable for helping decision-makers adjust climate pledges because NDCs reflect the intentions of parties regarding future climate actions, and parties are highly likely to follow the NDCs compared to SSPs and RCPs.

The reliability of forecasts of land cover/use maps depends on the accuracy of their baseline land cover/use maps in the starting year. Commonly used baseline land cover/use maps include Globeland30^20,21^, ESA-CCI^22^, and the MODIS Land Cover Type Product (MCD12Q1, https://lpdaac.usgs.gov/). The accuracy of these baseline land cover/use maps is limited^23,24^, leading to the reliability of forecasts of land cover/use maps being limited. Specifically, the accuracy of these baseline land cover/use maps varies across different regions and land cover/use types. For example, the accuracy of Globeland30 in 2010 ranged from 46.0% to 88.9%^25^. The accuracy of artificial surfaces exceeded 80%^21^, whereas the accuracy of wetlands was only 52.6%^25,26^ in Globeland30 in 2010. Additionally, only 8% of countries exhibit a cropland area difference of less than 1% between ESA-CCI and FAOSTAT^24^.

Additionally, existing forecasts of land cover/use maps that take into account NDCs neglect time series land cover/use changes. They generally forecasted land cover/use changes directly from the base year to 2100, which led to a lack of time series change details^4,17,18,27^. In particular, some studies suggest that land area changes under climate pledges do not follow a linear trajectory, but instead exhibit patterns of initial increase followed by decrease, or vice versa^4^. If time-series land cover/use changes are ignored, potential land cover/use changes under climate pledges may be underestimated. Such underestimation could lead to an inadequate assessment of associated risks, including biodiversity loss. Moreover, time series land cover/use change maps can better support decision-makers in adjusting land management policies. Specifically, near-term forecasts of land cover/use maps provide more actionable guidance, whereas long-term forecasts of land cover/use maps provide strategic insights for future planning.

In this study, we forecasted land cover/use changes in China from 2015 to 2100 at a 1 km resolution and 10-year interval (except for the 2015 to 2020 period) by integrating the GCAM model^2^, the Land-N2N model^28^, and the HIstoric Land Dynamics Assessment+ (HILDA+) land cover/use maps^29^. Here, we selected HILDA+^29^—the accurate land cover/use map—as our baseline land cover/use map. The high accuracy of HILDA + land cover/use maps stems from their harmonization of multiple available global, regional, and national land cover/use maps across multiple spatial resolutions^29^. By using the framework, we forecasted nine land cover/use maps in China under the 1.5 °C climate scenario that takes into account NDCs. The process of generating forecasts of land cover/use maps consisted of two main steps. First, we calculated the land demands using the GCAM. Second, we used the Land-N2N model to forecast the land cover/use change from 2015 to 2100 at a 10-year interval (except for 2015 to 2020 period). The Land-N2N model is effective and efficient in simulating spatially explicit changes. Additionally, the Land-N2N model can avoid the calibration of land areas in the GCAM because of the different land definitions for land cover/use maps and the GCAM. To demonstrate the effectiveness of the Land-N2N model, we evaluated the accuracy of the land cover/use changes from 2005 to 2015. In summary, the forecasts of land cover/use maps developed in this study offer essential data support and valuable insights for land management and adjustment of NDCs.

## Methods

### Overall framework

**Fig. 1:**
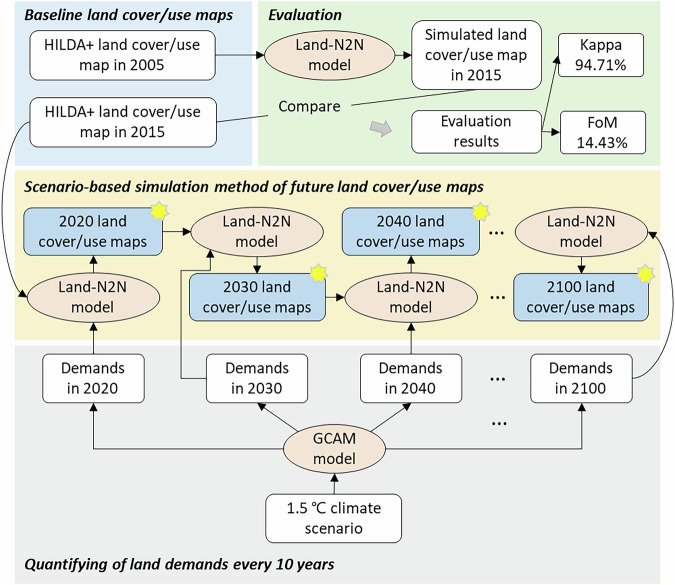
Overall framework. The asterisks indicate forecasts of land cover/use maps.

**Fig. 2:**
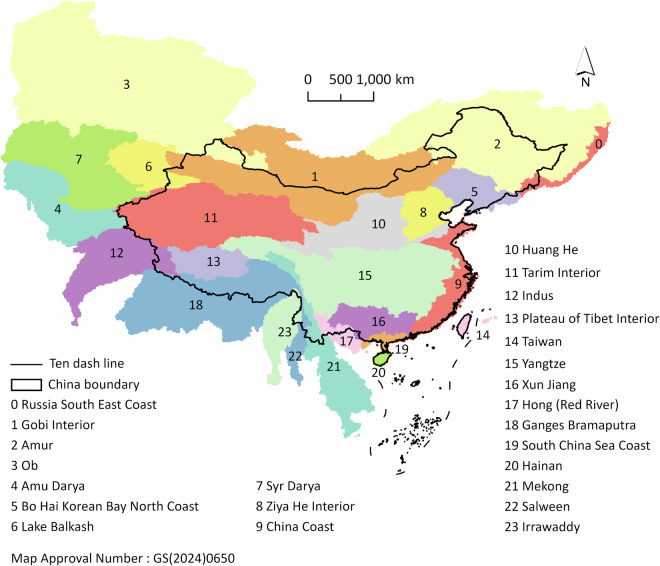
Study area and 24 corresponding water basins. The Chinese boundary was obtained at https://cloudcenter.tianditu.gov.cn/administrativeDivision/.

In this study, we proposed a framework that integrates the GCAM model, the Land-N2N model, and HILDA + land cover/use maps to forecast future land cover/use changes in China from 2015 to 2100 at a 10-year interval (except for the 2015 to 2020 period) and 1 km resolution. The overall framework is shown in Fig. 1. The framework was used in each water basin. In the GCAM model, the global world is divided into 235 water basins^30^. We selected China as the study area. On the basis of overlay analysis, 24 water basins overlap with the Chinese boundary (Fig. 2). For each basin, the simulation process consists of four steps. First, we selected HILDA + land cover/use maps in 2005 and 2015 to evaluate the framework, as these years align with the GCAM model outputs for evaluation. HILDA + land cover/use maps were also used to calibrate parameters for land cover/use change simulations. Second, we evaluated the performance of the framework by simulating land cover/use changes from 2005 to 2015. Third, we quantified future demands every 10 years (except for the 2015 to 2020 period) under the 1.5 °C climate scenario, which takes into account NDCs by using the GCAM model. Fourth, we used the Land-N2N model to forecast future land cover/use changes at a 10-year interval (except for the 2015 to 2020 period) at a 1 km resolution from 2015 to 2100.

### Selection of baseline land cover/use map and driving factors

In this study, we established three criteria for selecting a baseline land cover/use map. First, the temporal coverage of the maps should align with the calibration period of the GCAM model. Second, these maps should characterize historical land cover/use types as accurately as possible. Third, the spatial resolution must be approximately 1 km to align with the resolution of driving factors.

**Fig. 3:**
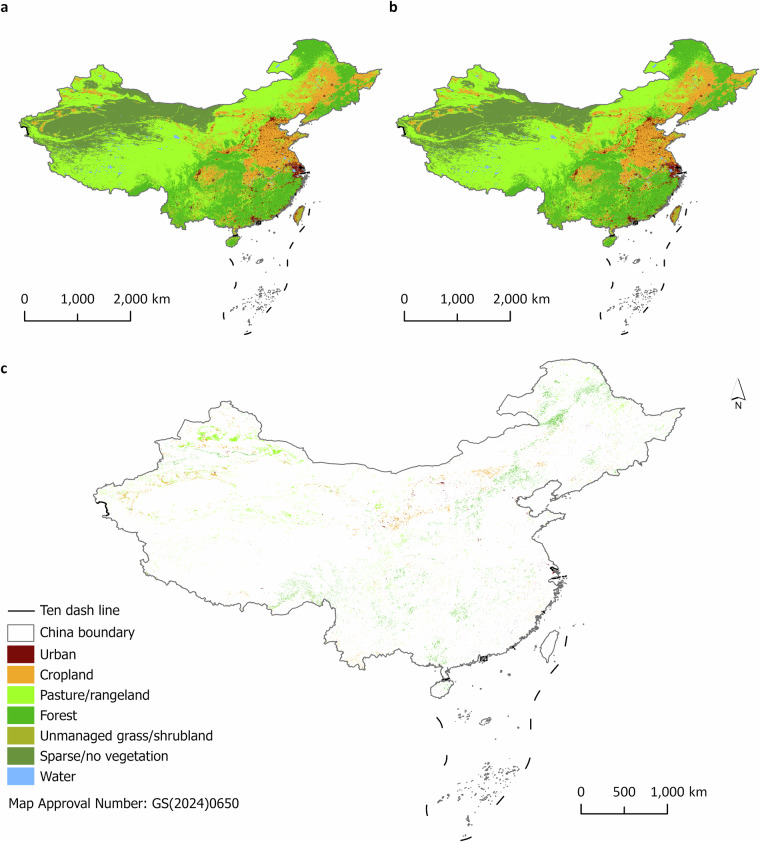
HILDA + land cover/use maps in China. The China boundary can be accessed at https://cloudcenter.tianditu.gov.cn/administrativeDivision/. **a**. HILDA + land cover/use map from 2005. **b**. HILDA + land cover/use map from 2015. **c**. Land cover/use changes from 2005 to 2015. The colorful areas indicate the land cover/use types in 2015 that experienced changes from 2005 to 2015. Blank areas indicate land cover/use types that remained unchanged from 2005 to 2015.

Based on these established criteria, we selected HILDA + land cover/use maps^29,31^ (https://doi.pangaea.de/10.1594/PANGAEA.921846) in 2015 as our baseline land cover/use maps. HILDA + land cover/use maps of China in 2005 and 2015 are shown in Fig. 3. HILDA + land cover/use maps consist of annual global land cover/use maps from 1960 to 2019 at a spatial resolution of 1 km. The HILDA + land cover/use maps consist of seven land cover/use types, namely urban, cropland, pasture/rangeland, forest, unmanaged grass/shrubland, sparse/no vegetation, and water. HILDA + land cover/use maps were generated using multiple types of Earth observation data and a variety of openly available global, continental, regional, and national land cover/use maps. Additionally, HILDA + land cover/use maps were calibrated using national land statistics provided by the Food and Agriculture Organization of the United Nations. Compared with other baseline land cover/use maps, HILDA + land cover/use maps demonstrate a greater ability to capture global land cover/use change^29^.

Driving factors are spatial natural, social, and economic factors that can influence land cover/use changes. In the Land-N2N model, driving factors were used to calculate a key parameter, local suitability. The selection of the driving factors was based on three criteria. First, the spatial resolution of the driving factors should be approximately 1 km to ensure consistency with the resolution of the baseline land cover/use map. Second, the temporal coverage of the driving factors should be centered around the year 2015 to align with the calibration period and baseline for forecasting. Third, the driving factors should comprehensively capture the dynamics of all land cover/use types as much as possible. Based on these three criteria, we selected seven categories of driving factors, following the methods of Gao *et al*.^4^. The seven categories of driving factors consist of soil, socio-economic, accessibility, agriculture/vegetation, terrain, climate, and livestock. The detailed driving factor list is shown in Table S1. In this study, we resampled all the driving factors into a 1 km resolution.

### Scenario-based simulation method for forecasts of land cover/use changes

In this study, we used the Land-N2N model^28^ to forecast land cover/use changes under the 1.5 °C climate scenario that takes into account NDCs. The Land-N2N model is capable of simulating spatially explicit land cover/use changes in response to changes in all demands. The advantage of the Land-N2N model lies in its flexibility in establishing relationships between demands and land cover/use types. This advantage enables the Land-N2N model to integrate more conveniently with the GCAM model, avoiding the need for calibrations of the GCAM model due to discrepancies in land cover/use type definitions between the baseline land cover/use map and the GCAM model.

**Fig. 4:**
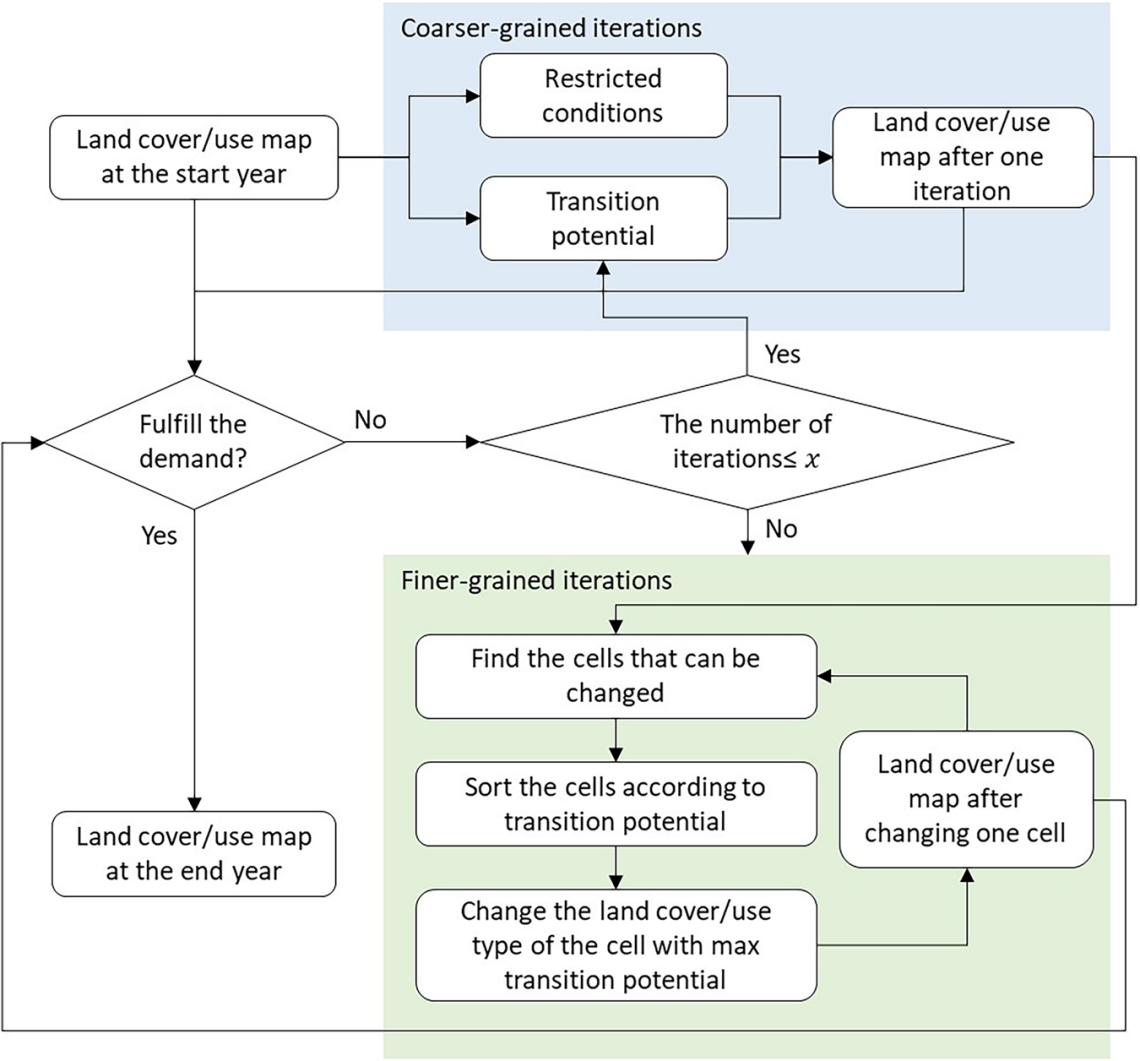
Iteration mechanism in the Land-N2N model. *x* indicates the number of coarser-grained iterations, and is assigned by users.

In the Land-N2N model, land cover/use maps are forecasted through multiple iterations^28^. At the end of each iteration, the Land-N2N model assesses whether the supply‒demand balance has been achieved. If a supply‒demand balance is achieved, the Land-N2N model concludes the iteration process. Compared to other land cover/use change models, the Land-N2N model employs a combined iteration mechanism that consists of coarser-grained iterations and finer-grained iterations to help the model generate simulation results (Fig. 4). The coarser-grained iterations are designed to quickly approach the approximate balance between supplies and demands. If the coarser-grained iterations fail to achieve a supply‒demand balance, the finer-grained iterations will be implemented to achieve supply‒demand balances cell-by-cell. In this study, Table S2 shows the average and maximum differences between demands and supplies for each water basin.

In each iteration, the land cover/use type of each cell was changed on the basis of land cover/use change rules. The land cover/use change rules consisted of the transition potential and restricted conditions. The transition potential represents the likelihood of a cell changing to each land cover/use type. The transition potential is calculated as the sum of local suitability, competitive advantages, resistance, and neighborhood effects. Local suitability is the only spatially explicit parameter, representing the likelihood of change for each land cover/use type driven by driving factors. Competitive advantages reflect whether the area of each land cover/use type should increase or decrease to fulfill all demands. Resistances reflect the difficulties of changing to other land cover/use types. Neighborhood effects represent the effect of adjacent cells on the land cover/use type of the center cell. The restricted conditions consist of spatially restricted regions and a conversion matrix. The spatially restricted regions specify areas where land cover/use remains unchanged. The conversion matrix defines whether conversion between two land cover/use types is permissible. The detailed calculation methods for each parameter and the restricted conditions are as follows.

#### Local suitability

In the Land-N2N model, local suitability was calculated using random forest models^28^. The random forest model is a machine-learning algorithm widely used for both classification and regression^32,33^. In this study, we established the complex relationships between land cover/use changes and the driving factors for each land cover/use type in each water basin. For each land cover/use type, we created a random forest model using 200 decision trees.

The random forest models were trained on samples consisting of positive and negative samples. Each sample consists of a label and a set of driving factors. For each land cover/use type, we generated positive and negative samples using land cover/use maps from 2005 and 2015, along with driving factors. For the *i*-th land cover/use type, if the land cover/use type of a cell was changed to the *i*-th land cover/use type from other land cover/use types from 2005 to 2015, we labeled the cell and corresponding driving factors as a positive sample. Otherwise, we labeled the cell and corresponding driving factors as a negative sample.

For each land cover/use type, 25% of the samples were used to train the random forest model, whereas the remaining 75% were used to evaluate the performance of the random forest model using the area under the curve (AUC). The AUC ranges from 0 to 1, with higher values indicating better performance of the random forest model. The AUC of 24 water basins for each land cover/use type is shown in Table S3.

#### Competitive advantages

In the Land-N2N model, competitive advantages represent whether a land cover/use type should increase or decrease to fulfill the demands we have specified. In each iteration, competitive advantages are adjusted according to differences between supplies and demands and the capacity of each land cover/use type to fulfill demands. If a demand has not yet been fulfilled, the competitive advantage of the land cover/use type with a higher capacity to fulfill demand will be increased in the next iteration. Conversely, if a certain demand has already been exceeded, the competitive advantage of the land cover/use type with a lower capacity to fulfill demand will be decreased in the next iteration.

#### Supply capacity

In the Land-N2N model, supply capacities represent the capacity of each land cover/use type to fulfill demands. To quantify the supply capacities, we established one-to-one relationships between land cover/use types and demands (the method for quantifying the four demands is described in the section “Quantification of land demands every 10 years under 1.5 °C climate”). Specifically, we assumed that the areas of cropland, pasture/rangeland, forest, and unmanaged grass/shrubland in the GCAM model are served by cropland, pasture/rangeland, forest, and unmanaged grass/shrubland cells in the land cover/use maps, respectively. The supply capacities for cropland, pasture/rangeland, forest, and unmanaged grass/shrubland types are calculated by dividing the areas from the GCAM model by the number of cells in the land cover/use maps. We assumed that urban, sparse/no vegetation, and water cannot fulfill any demands.

#### Resistances

In the Land-N2N model, the resistances range from 0 to 1. A higher value indicates that one land cover/use type is more difficult to change to other land cover/use types. In this study, the resistances were calculated using overlay analysis based on HILDA + land cover/use maps of each water basin in 2005 and 2015. Specifically, the resistance of each land cover/use type was calculated as the proportion of that land cover/use type that remained unchanged from 2005 to 2015.

#### Conversion matrix

In the Land-N2N model, the conversion matrix is composed of binary values, namely, ‘0’ and ‘1’. Here, ‘0’ indicates that the conversion from one land cover/use type to another is not permitted, whereas ‘1’ indicates that the conversion is permitted. In the study, we calculated the conversion matrix according to HILDA + land cover/use maps of each water basin in 2005 and 2015. Specifically, if the *i*-th land cover/use type was changed to the *j*-th land cover/use type from 2005 to 2015, we would allow the *i*-th land cover/use type to change to the *j*-th land cover/use type. In contrast, we would not allow the *i*-th land cover/use type to change to the *j*-th land cover/use type. Importantly, we assumed that urban and water will remain unchanged in the future.

#### Neighborhood effects

In the Land-N2N model, neighborhood effects are determined by the neighborhood size, neighborhood weight, and land cover/use types that influence the center cell. In this study, we assigned a neighborhood size is 3 × 3 cells. The center cell is influenced only by the land cover/use type, which is the same as the center cell. The neighborhood weight ranges from 0 to 1. The detailed neighborhood weights for each land cover/use type in each water basin are shown in Table S4.

#### Restricted regions

In this study, no spatially restricted regions were defined.

### Quantification of land demands every 10 years under 1.5 °C climate

We quantified the land demands every 10 years (except for the 2015 to 2020 period) under the 1.5 °C climate scenario that takes into account NDCs by using the GCAM model^2^. The GCAM model is a global market equilibrium model^30^. The GCAM model consists of five modules, namely water, energy, climate, socioeconomic, and land. The land module of the GCAM model can generate land areas from 1990 to 2100 at a five-year interval for each water basin. The land areas are regarded as the demand in the land change simulations.

In this study, we selected the seventh scenario in the study of Iyer *et al*.^2^ as the 1.5 °C climate scenario. Specifically, Iyer *et al*.^2^ explored the final temperature change and the peak temperature change before 2100 under 27 scenarios. These scenarios considered various decarbonization rates (2%, 5%, and 8%), different ambition levels (“NDC”, “NDC+”, and “NDC++”), and three timelines for achieving net-zero emissions (on schedule, five years earlier, and 10 years earlier). In the GCAM model, NDCs are implemented through absolute emissions limit, percentage emission reductions from a given reference level, and emission intensity targets^2^. For the “NDC+” level, parties rated “critically insufficient” and “highly insufficient” by Climate Action Tracker (CAT) are assumed to reduce their emissions by 30% below their current NDCs. For the “NDC++” level, parties rated “critically insufficient” and “highly insufficient” by CAT are assumed to reduce their emissions by 30% below their current NDCs. The seventh scenario, namely the 1.5 °C climate scenario in our study, shows that the final temperature change can be kept below 1.5 °C, with the peak temperature change reaching approximately 1.8 °C. This scenario assumes that all parties would adhere to their submitted NDCs, namely, the ambition level is “NDC”. This scenario also assumes that the decarbonization rate in the 1.5 °C climate scenario is 8% after 2030 and that the timeline for achieving net-zero emissions is on schedule.

We quantified land demands every 10 years (except for the 2015 to 2010 period) by regrouping land types in the GCAM model under the 1.5 °C climate scenario. The GCAM model consists of 70 land types. Among the 70 land types, three land types remained unchanged, namely tundra, rock-ice-desert, and urban. Therefore, we excluded these three land types when we regrouped land types in the GCAM model. We regrouped the remaining 67 land types into four demands, namely the areas of cropland, pasture/rangeland, forest, and unmanaged grass/shrubland. The corresponding relationships between demands and land types in the GCAM model are shown in Table 1. It is important to note that although the demands here are expressed as the areas of land types in GCAM, we do not treat them as physical areas, but rather as services that drive changes in land cover/use.

**Table 1:** Relationships between demands and land types in the GCAM model.

Demands	Land types in GCAM
Cropland	Corn_IRR/RFD_hi/lo
	FiberCrop_IRR/RFD_hi/lo
	FodderGrass_IRR/RFD_hi/lo
	FodderHerb_IRR/RFD_hi/lo
	MiscCrop_IRR/RFD_hi/lo
	OilCrop_IRR/RFD_hi/lo
	PalmFruit_IRR/RFD_hi/lo
	Rice_IRR/RFD_hi/lo
	RootTuber_IRR/RFD_hi/lo
	SugarCrop_IRR/RFD_hi/lo
	Wheat_IRR/RFD_hi/lo
	BiomassGrass_IRR/RFD_hi/lo
	BiomassTree_IRR/RFD_hi/lo
	OtherGrain_IRR/RFD_hi/lo
	OtherArableLand
Pasture/rangeland	Pasture
	UnmanagedPasture
	ProtectedUnmanagedPasture
Forest	Forest
	UnmanagedForest
	ProtectedUnmanagedForest
Unmanaged grass/shrubland	GrassLand
	ProtectedGrassland
	ShrubLand
	ProtectedShrubland

The cropland types in the GCAM model are defined with the irrigation types and fertilizer levels. Here, “IRR” indicates “irrigated”, “RFD” indicates “rainfed”, “hi” indicates “high fertilizer”, and “lo” indicates “low fertilizer”.

## Data Records

**Fig. 5:**
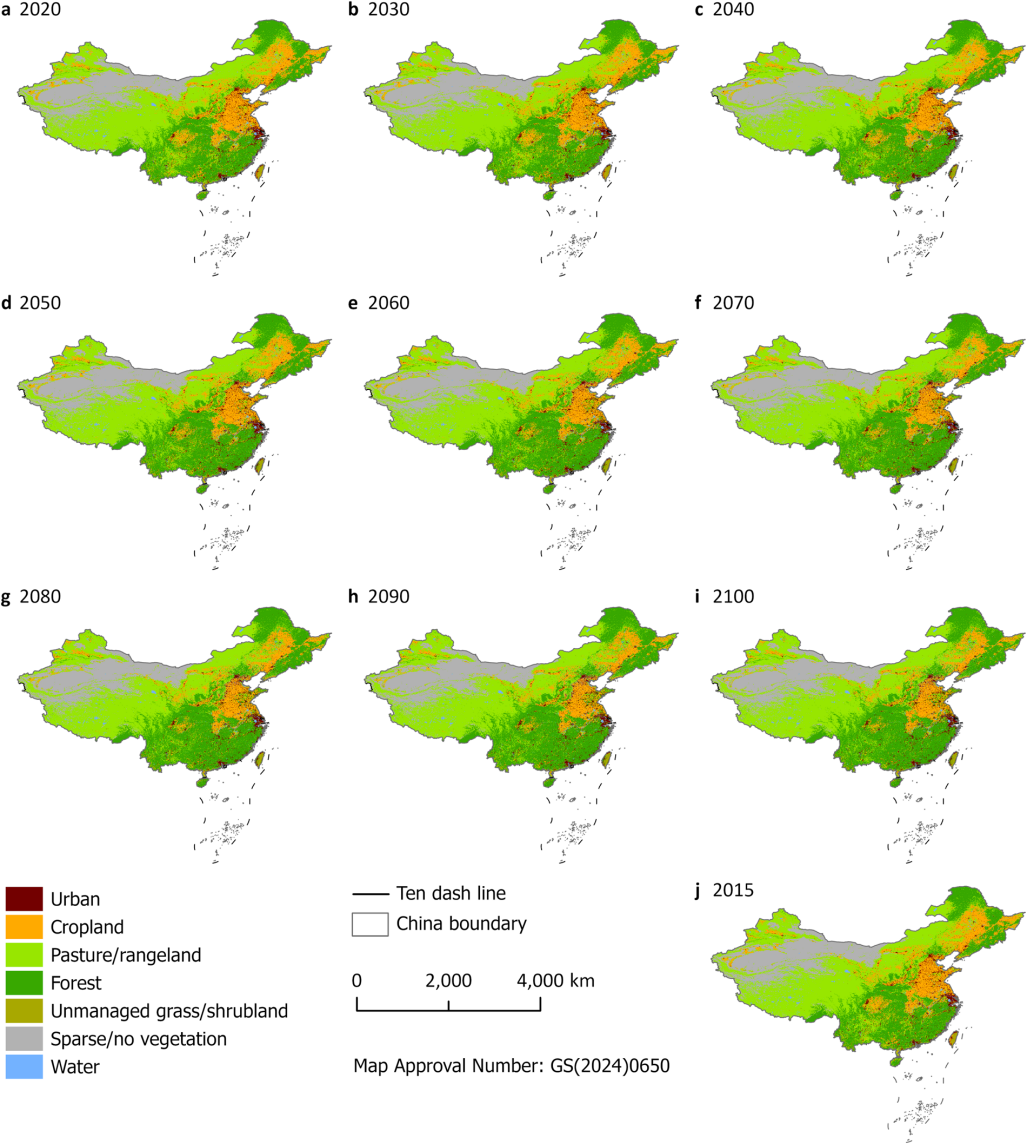
Land cover/use maps from 2015 to 2100 under the 1.5 °C climate scenario. The China boundary can be accessed at https://cloudcenter.tianditu.gov.cn/administrativeDivision/.

**Fig. 6:**
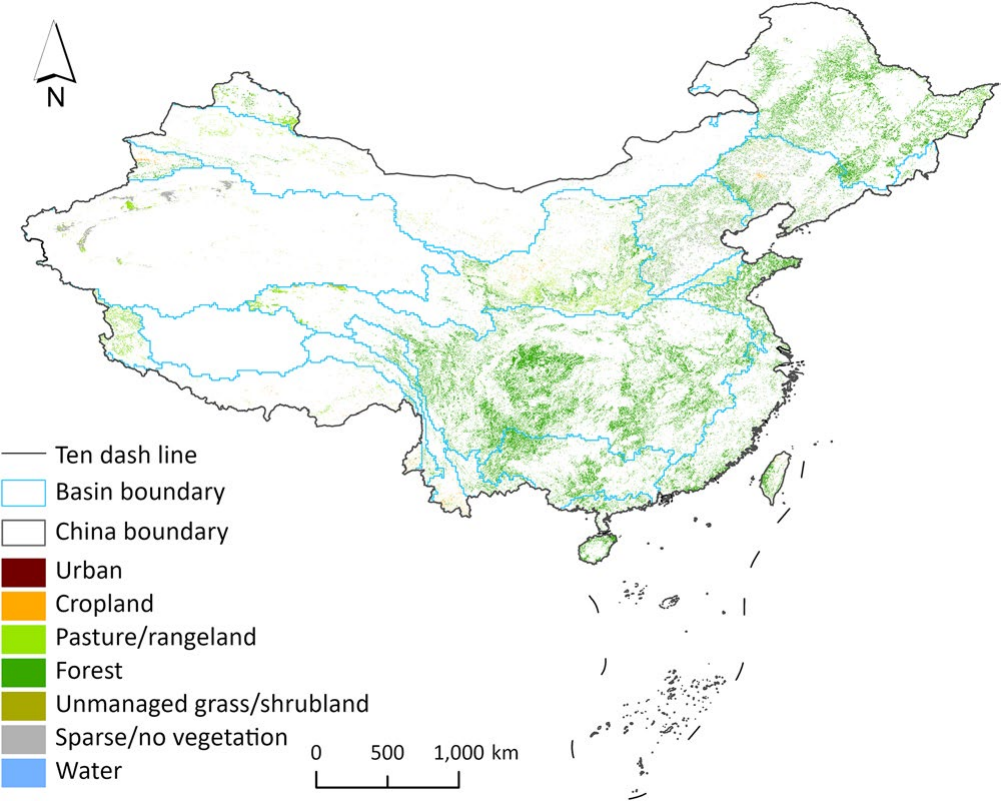
Land cover/use change maps under the 1.5 °C climate scenario from 2015 to 2100. The China boundary can be accessed at https://cloudcenter.tianditu.gov.cn/administrativeDivision/. The colorful regions indicate areas where the land cover/use type changes, whereas the blank regions indicate areas where the land cover/use type remains unchanged.

The dataset is available at Figshare^34^. The dataset consists of 10 land cover/use maps of China at 1 km resolution under the 1.5 °C climate scenario that takes into account NDCs (Fig. 5). Additionally, Fig. 6 shows the changes in land cover/use from 2015 to 2100. The format for naming these land cover/use maps is “lu_Year.tif”. In the file name, “Year” indicates the year of the land cover/use map. These land cover/use maps are stored in GeoTIFF format (.tif). We projected these land cover/use maps into the Cylindrical Equal Area (world). In these land cover/use maps, we used integers from 0 to 6 to assign land cover/use types. These integers represent urban, cropland, pasture/rangeland, forest, unmanaged grass/shrubland, sparse/no vegetation, and water, respectively. To facilitate visualization, we also provided a color map file (named “color.clr”), which can be directly used in ArcGIS or ArcGIS Pro.

## Technical Validation

### Evaluation and calibration methods for framework application in China

In this study, we evaluated and calibrated the application of the framework in China by simulating land cover/use changes in each water basin from 2005 to 2015. The purpose of calibrating is to estimate the suitability of parameters. The key assumption underlying our evaluation methods is that if the framework is able to effectively simulate the historical land cover/use changes, the framework is able to effectively forecast future land cover/use changes, and the parameters are suitable. In this study, we evaluated the framework using the Kappa coefficient^35^ and the Figure of Merit (FoM)^36^, which assess the framework’s performance from two perspectives. The Kappa coefficient is used to evaluate the agreement between the simulated land cover/use map and the actual land cover/use map, accounting for the agreement caused by random chance. The Kappa coefficient primarily reflects spatial stability rather than dynamic change. In contrast, the FoM aims to evaluate the change accuracy^36^. FoM is specifically designed to capture dynamic changes by focusing exclusively on the cells that changed during the simulation. The Kappa coefficient and the FoM are calculated through Eqs. (1) and (2). The Kappa coefficient ranges from −1 to 1^37^. If the value of the Kappa coefficient is less than 0, it indicates that the simulation accuracy is worse than random chance. Conversely, the value of the Kappa coefficient closer to 1 represents greater agreement between the simulated and actual land cover/use maps, reflecting greater simulation accuracy. The FoM ranges from 0 to 1, with higher values indicating greater simulation accuracy.

1 $${\rm{Kappa}}=\frac{{p}_{0}-{p}_{c}}{1-{p}_{c}}$$

2 $${\rm{FoM}}=\frac{B}{A+B+C+D}$$

Here, *p*_0_ represents the observed agreement between the actual and simulated land cover/use maps. *p*_*c*_ represents the agreement between the actual land cover/use map and the simulated land cover/use map caused by random changes. In Eq.(2), *A* represents the error area where the actual land cover/use change is incorrectly simulated as unchanged. *B* represents the accurate area where the actual land cover/use change is correctly simulated as a change. *C* represents the error area where the actual change is simulated as a change, but as an incorrect land cover/use type. *D* represents the error area where actual unchanged values are simulated as changes.

If the calibrated parameters, including resistance, neighborhood effect, and conversion matrix, did not constrain forecasts, we used them directly to forecast land cover/use changes. But if they constrained forecasts, we adjusted them accordingly. For example, if the forecasts required forest expansion but the conversion matrix did not allow forest expansion, we would adjust the conversion matrix to allow forest expansion. Furthermore, if the demand changes simulated by GCAM show a clear contrast with those observed in HILDA + during the framework evaluation, the demand parameters are recalibrated to ensure consistency.

### Accuracy evaluations of the framework application in China

**Fig. 7:**
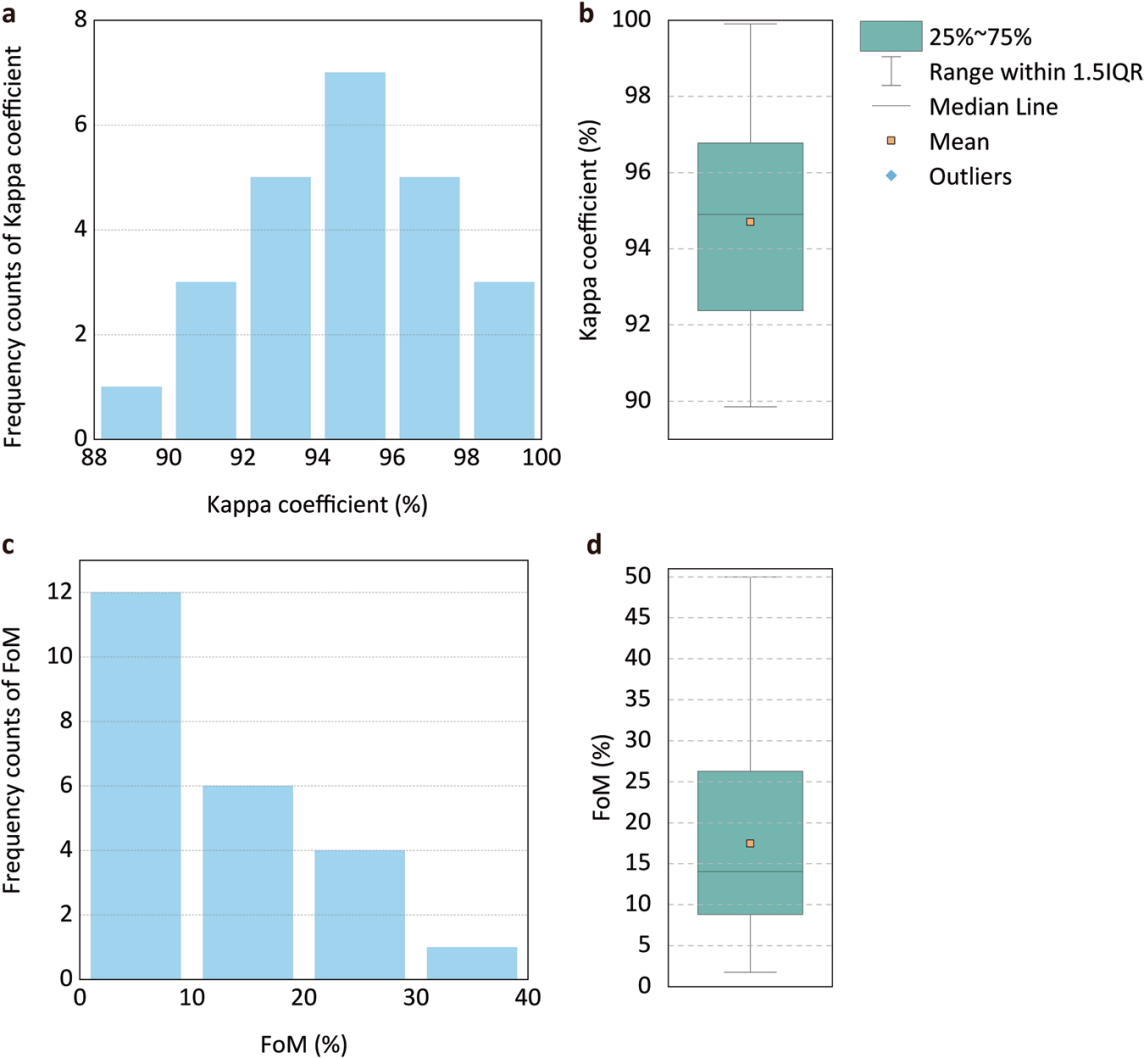
Distribution of Kappa coefficients and FoM in 24 water basins. **a.** Histogram of Kappa coefficients. **b**. Box plot of Kappa coefficients. **c**. Histogram of FoMs. **d**. Box plot of FoMs.

On the basis of the Kappa coefficients and FoM for 24 water basins, the framework applied in China demonstrates the ability to effectively forecast land cover/use changes and low uncertainty in simulations. Specifically, the calculated Kappa coefficients and FoM are shown in Table 2. The average Kappa coefficient is 94.71%. The average FoM is 14.43%. The distribution of Kappa coefficients and FoM is shown in Fig. 7. The median of the Kappa coefficients is 94.85%. The median FoM is 10.39%. Kappa coefficients across the five water basins range from 96% to 98%, whereas the FoM in 18 water basins exceeds 10%.

**Table 2:** Kappa coefficients and FoMs for 24 water basins.

Water basin	Kappa	FoM	Water basin	Kappa	FoM
Russia South East Coast	92.42%	4.17%	Indus	94.85%	12.05%
Gobi Interior	95.05%	5.67%	Plateau of Tibet Interior	98.95%	18.76%
Amur	94.27%	3.01%	Taiwan	99.90%	50.00%
Ob	98.39%	8.70%	Yangtze	97.39%	25.84%
Amu Darya	95.88%	3.73%	Xun Jiang	97.94%	26.27%
Bo Hai Korean Bay North Coast	93.76%	6.87%	Hong (Red River)	89.85%	18.41%
Lake Balkash	90.80%	8.03%	Ganges Bramaputra	93.58%	8.80%
Syr Darya	92.18%	5.47%	South China Sea Coast	96.48%	28.69%
Ziya He Interior	90.27%	4.14%	Hainan	96.52%	27.23%
China Coast	97.03%	16.00%	Mekong	92.34%	31.33%
Huang He	94.17%	10.39%	Salween	94.95%	11.08%
Tarim Interior	95.37%	1.76%	Irrawaddy	90.65%	9.93%

### Comparison with other land cover/use maps for the Kappa coefficient and FoM

Compared with other land cover/use maps, the Kappa coefficients and FoM in this study demonstrated that the framework applied in China is suitable for forecasting land cover/use changes. The Kappa coefficients and FoMs of other similar land cover/use maps are shown in Table 3. Specifically, the average Kappa coefficients in the study exceed all Kappa coefficients of other similar land cover/use maps listed in Table 3. In particular, the lowest Kappa coefficient (89.85%, in Hong (Red River)) among the 24 water basins exceeds all the Kappa coefficients of other similar land cover/use maps listed in Table 3. The FoM in this study is comparable to that reported in the studies of Table 3. Among the 24 water basins, the FoM of 11 water basins exceeded 10%.

**Table 3:** Kappa coefficients and FoMs of other similar land cover/use maps.

Land cover/use maps	Region	Thematic resolution	Kappa	FoM
Luo *et al*.^13^	China	8	[43%, 75%]	[10%, 17%]
Li *et al*.^38^	East Asia	6	—	28.25%
Liu *et al*.^36^	China	6	67%	19.62%
Lv *et al*.^18^	China	27	81.27%	17.74%
Lin *et al*.^39^	China	6	81%	17%
Wang *et al*.^40^	China	27	84%	—

In the table, the thematic resolution refers to the number of types^41,42^. “-” indicates that the land cover/use maps were not evaluated through the Kappa coefficient or FoM. The decimal values in the table are dependent on the decimal reported in the original paper.

### Comparison with another land system map for spatial patterns

**Fig. 8:**
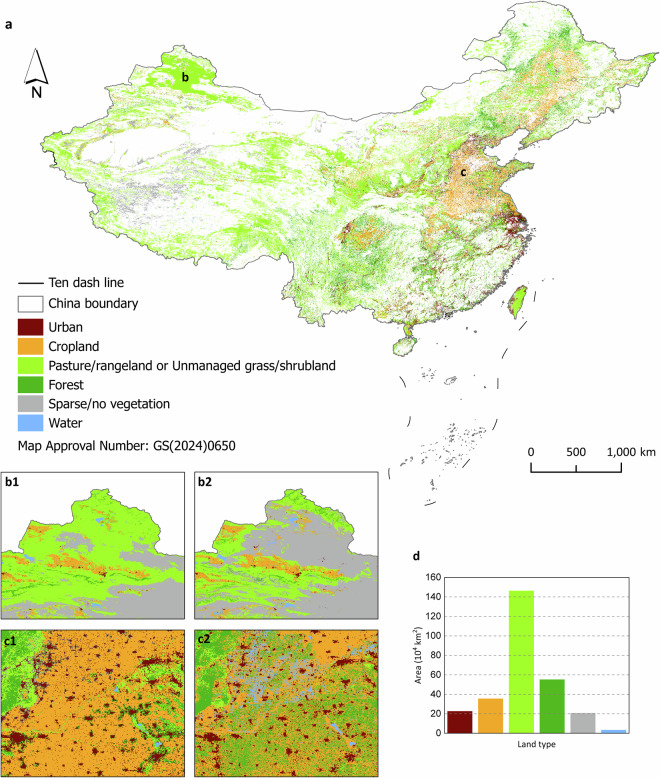
Comparison of the 2100 land cover/use map used in this study (named Map LU) and the 2100 land system map provided by Lv, *et al*.^18^ (Map LS). The China boundary can be accessed at https://cloudcenter.tianditu.gov.cn/administrativeDivision/. **a**. Comparison map between Map LU and Map LS after reclassifications. The colorful regions represent the land cover/use type in Map LU after reclassification, where the land cover/use type is different between the two maps. The blank regions represent the regions where the land cover/use type after reclassification is the same between the two maps. **b1-b2**. The land cover/use type after reclassification in Map LU (b1) and Map LS (b2) at the junction of the Ob and Gobi Interior. **c1-c2**. The land cover/use type after reclassification in Map LU (c1) and Map LS (c2) in the Ziya He Interior. **d**. Difference in areas of land use types in the two maps.

In this study, we compared the 2100 land cover/use map with the 2100 land system map provided by Lv *et al*.^18^ to highlight the importance of using more accurate baseline land cover/use maps. It should be noted that both maps were forecasted under the same scenario. The comparison demonstrated that the advantage of our land cover/use maps is that they are more reliable because they used more accurate baseline land cover/use maps. Owing to differences in thematic resolution between the two land maps, we reclassified both the 2100 land system map provided by Lv *et al*.^18^ and the 2100 land cover/use map forecasted in the study. The reclassification rules are shown in Table 4. The comparison map is shown in Fig. 8. According to cell-by-cell analysis, we found a 70.15% agreement in the spatial patterns between the two maps. The largest discrepancy is observed in the pasture/rangeland and unmanaged grass/ shrubland types, which account for 15.42% of the difference (146 × 10^4^ km^2^). For example, at the junction of the Ob and Gobi Interior, the pasture/rangeland and unmanaged grass/shrubland types in our 2100 land cover/use map were forecasted as sparse/no vegetation in the 2100 land system map provided by Lv *et al*.^18^ (Fig. 8b1, b2). A secondary discrepancy was observed in the forest, accounting for 5.81% of the differences (55 × 10^4^ km^2^). Additionally, we found that the cropland in the 2100 land system map provided by Lv *et al*.^18^ is more fragmented than the cropland in our 2100 land cover/use map (Fig. 8c1, c2).

**Table 4:** Reclassification rules for 2100 land system maps.

Land cover/use type	Land system type
Urban	Low density artificial surfaces
	Medium density artificial surfaces
	High density artificial surfaces
Cropland	Low density cropland
	Medium density cropland
	High density cropland
Pasture/rangeland and unmanaged grass/shrubland	Low density shrubland
	Medium density shrubland
	High density shrubland
	Low density grassland
	Medium density grassland
	High density grassland
Forest	Low density forest
	Medium density forest
	High density forest
Sparse/no vegetation	Low density wetland
	Medium density wetland
	High density wetland
	Low density bareland
	Medium density bareland
	High density bareland
	Low density snow and ice
	Medium density snow and ice
	High density snow and ice
Water	Low density water bodies
	Medium density water bodies
	High density water bodies

## Usage Notes

In this study, we forecasted nine 1 km land cover/use maps of China under the 1.5 °C climate scenario, taking into account NDCs, from 2015 to 2100 at a 10-year interval (except for the 2015 to 2010 period) by integrating the Land-N2N model, the GCAM model, and HILDA + land cover/use maps. Compared with existing forecasts of land cover/use maps under the 1.5 °C climate scenario, there are three advantages. First, we used more accurate land cover/use maps as the baseline land cover/use map to increase the reliability of forecasts of land cover/use maps. Second, these maps are able to capture detailed information on land cover/use changes over time. Third, we forecasted land cover/use changes at a 1 km resolution. Compared with the land system map (5 km) forecasted in the study of Gao *et al*.^19^ under the 1.5 °C climate scenario, the spatial resolution (1 km) of the land cover/use maps in this study is greater. To evaluate the integrated framework, we used the Kappa coefficient and FoM to simulate land cover/use changes from 2005 to 2015. The calculation results of the Kappa coefficient and FoM indicate that the integrated framework is able to forecast land cover/use change effectively. In addition, we also acknowledge that the forecasts of land cover/use maps have certain limitations. Specifically, our assumption of unchanged urban areas makes the maps unsuitable for applications that focus on urban expansion.

## Supplementary information

Supplementary Information of 1 km HILDA+ based land cover/use map time series of China under 1.5 °C climate of this century

## Data Availability

The dataset is available at Figshare^34^ (10.6084/m9.figshare.29590499). The dataset consists of ten land cover/use maps of China at 1 km resolution under the 1.5 °C climate scenario that takes into account NDCs. A detailed description of the dataset is provided in the Data Record section.

## Code availability

In this study, we used the Land-N2N model to forecast land cover/use changes. The codes of the Land-N2N model can be accessed at https://github.com/gaoyifan2021/Land-N2N-v1.

## References

[1] Ou, Y. *et al*. Can updated climate pledges limit warming well below 2 °C? *Science* **374**, 693–695 (2021). 34735225 10.1126/science.abl8976

[2] Iyer, G. *et al* Ratcheting of climate pledges needed to limit peak global warming. *Nature Climate Change*, 1–7 (2022). 10.1038/s41558-022-01508-0 PMC10569109 37829842

[3] Savin, I., King, L. C., van den Bergh, J. Analysing content of Paris climate pledges with computational linguistics. *Nature Sustainability*, 1–10 (2025).

[4] Gao, P. *et al* Fulfilling global climate pledges can lead to major increase in forest land on Tibetan Plateau. *iScience* **26** (2023). 10.1016/j.isci.2023.106364 PMC10060679 37009210

[5] Roe, S. *et al*. Contribution of the land sector to a 1.5 C world. *Nature Climate Change* **9**, 817–828 (2019).

[6] Searchinger, T. D., Wirsenius, S., Beringer, T. & Dumas, P. Assessing the efficiency of changes in land use for mitigating climate change. *Nature* **564**, 249–253 (2018). 30542169 10.1038/s41586-018-0757-z

[7] Qin, Z. *et al*. Global spatially explicit carbon emissions from land-use change over the past six decades (1961–2020). *One Earth* **7**, 835–847 (2024).

[8] Qiu, L., He, J., Yue, C., Ciais, P. & Zheng, C. Substantial terrestrial carbon emissions from global expansion of impervious surface area. *Nature Communications* **15**, 6456 (2024). 39085270 10.1038/s41467-024-50840-w PMC11291968

[9] Zhao, Q., Yu, L. & Chen, X. Land system science and its contributions to sustainable development goals: A systematic review. *Land Use Policy* **143**, 107221 (2024).

[10] Zhu, Y. *et al* China’s carbon sinks from land-use change underestimated. *Nature Climate Change*, 1–8 (2025).

[11] Brovkin, V. *et al*. Role of land cover changes for atmospheric CO2 increase and climate change during the last 150 years. *Global Change Biology* **10**, 1253–1266 (2004).

[12] Popp, A. *et al*. Land-use protection for climate change mitigation. *Nature Climate Change* **4**, 1095–1098 (2014).

[13] Luo, M. *et al*. 1 km land use/land cover change of China under comprehensive socioeconomic and climate scenarios for 2020–2100. *Scientific data* **9**, 110 (2022). 35347153 10.1038/s41597-022-01204-w PMC8960815

[14] Zhang, T., Cheng, C. & Wu, X. Mapping the spatial heterogeneity of global land use and land cover from 2020 to 2100 at a 1 km resolution. *Scientific Data* **10**, 748 (2023). 37898602 10.1038/s41597-023-02637-7 PMC10613310

[15] Chen, G., Li, X. & Liu, X. Global land projection based on plant functional types with a 1-km resolution under socio-climatic scenarios. *Scientific Data* **9**, 125 (2022). 35354830 10.1038/s41597-022-01208-6 PMC8967933

[16] Chen, G. *et al*. Global projections of future urban land expansion under shared socioeconomic pathways. *Nature communications* **11**, 537 (2020). 31988288 10.1038/s41467-020-14386-x PMC6985221

[17] Lv, J. *et al*. Land system changes of terrestrial tipping elements on Earth under global climate pledges: 2000–2100. *Scientific Data* **12**, 163 (2025). 39870678 10.1038/s41597-025-04444-8 PMC11772770

[18] Lv, J., Song, C., Gao, Y., Ye, S., Gao, P. Simulation and analysis of the long-term impacts of 1.5 °C global climate pledges on China’s land systems. *Science China Earth Sciences*, 1–16 (2025).

[19] Gao, P. *et al* Heterogeneous pressure on croplands from land-based strategies to meet the 1.5 °C target. *Nature Climate Change* (2025).

[20] Chen, J., Ban, Y. & Li, S. Open access to Earth land-cover map. *Nature* **514**, 434–434 (2014). 10.1038/514434c 25341776

[21] Chen, J. *et al*. Global land cover mapping at 30 m resolution: A POK-based operational approach. *ISPRS Journal of Photogrammetry and Remote Sensing* **103**, 7–27 (2015).

[22] Bontemps, S. *et al* in *Proceedings of the ESA living planet symposium*. 9–13.

[23] Wang, Y. *et al*. Evaluation of six global high-resolution global land cover products over China. *International Journal of Digital Earth* **17**, 2301673 (2024).

[24] Liu, X. *et al*. Comparison of country-level cropland areas between ESA-CCI land cover maps and FAOSTAT data. *International Journal of Remote Sensing* **39**, 6631–6645 (2018).

[25] Liu, L. *et al* Finer-resolution mapping of global land cover: Recent developments, consistency analysis, and prospects. *Journal of Remote Sensing* (2021).

[26] Zhang, X. *et al*. GLC_FCS30: Global land-cover product with fine classification system at 30 m using time-series Landsat imagery. *Earth System Science Data Discussions* **2020**, 1–31 (2020).

[27] Gao, Y. *et al* Global land system maps at 1 km resolution for 1.5 °C climate. *Scientific Data* **12** (2025). 10.1038/s41597-025-04991-0 PMC12015323 40263311

[28] Gao, Y., Song, C., Liu, Z., Ye, S. & Gao, P. Land-N2N: An effective and efficient model for simulating the demand-driven changes in multifunctional lands. *Environmental Modelling & Software* **185**, 106318 (2025).

[29] Winkler, K., Fuchs, R., Rounsevell, M. & Herold, M. Global land use changes are four times greater than previously estimated. *Nature communications* **12**, 2501 (2021). 33976120 10.1038/s41467-021-22702-2 PMC8113269

[30] Calvin, K. *et al*. GCAM v5. 1: representing the linkages between energy, water, land, climate, and economic systems. *Geoscientific Model Development* **12**, 677–698 (2019).

[31] Winkler, K., Fuchs, R., Rounsevell, M. D. A. & Herold, M. HILDA+ Global Land Use Change between 1960 and 2019. *PANGAEA*10.1594/PANGAEA.921846 (2020).

[32] Breiman, L. Random forests. *Machine learning* **45**, 5–32 (2001).

[33] Lv, J. *et al*. Simulating urban expansion by incorporating an integrated gravitational field model into a demand-driven random forest-cellular automata model. *Cities* **109**, 103044 (2021).

[34] Gao, Y. *et al*. 1 km HILDA+ based land cover/use map time series of China under 1.5 °C climate of this century. *figshare*10.6084/m9.figshare.29590499 (2025). 10.1038/s41597-025-06411-9 PMC12830754 41381511

[35] Cohen, J. A coefficient of agreement for nominal scales. *Educational and psychological measurement* **20**, 37–46 (1960).

[36] Liu, X. *et al*. A future land use simulation model (FLUS) for simulating multiple land use scenarios by coupling human and natural effects. *Landscape and Urban Planning* **168**, 94–116 (2017).

[37] van Vliet, J., Bregt, A. K. & Hagen-Zanker, A. Revisiting Kappa to account for change in the accuracy assessment of land-use change models. *Ecological modelling* **222**, 1367–1375 (2011).

[38] Li, X. *et al*. A new global land-use and land-cover change product at a 1-km resolution for 2010 to 2100 based on human–environment interactions. *Annals of the American Association of Geographers* **107**, 1040–1059 (2017).

[39] Lin, X. *et al*. Projecting diversity conflicts of future land system pathways in China under anthropogenic and climate forcing. *Earth’s Future* **11**, e2022EF003406 (2023).

[40] Wang, Y., Song, C., Gao, Y., Ye, S. & Gao, P. Integrating national integrated assessment model and land-use intensity for estimating China’s terrestrial ecosystem carbon storage. *Applied Geography* **162**, 103173 (2024).

[41] García-Álvarez, D., Lloyd, C. D., Van Delden, H. & Olmedo, M. T. C. Thematic resolution influence in spatial analysis. An application to Land Use Cover Change (LUCC) modelling calibration. *Computers, environment and urban systems* **78**, 101375 (2019).

[42] Van der Biest, K. *et al*. Evaluation of the accuracy of land-use based ecosystem service assessments for different thematic resolutions. *Journal of environmental management* **156**, 41–51 (2015). 25794965 10.1016/j.jenvman.2015.03.018

