# Supplementary material for: 1 km HILDA + based land cover/use map time series of China under 1.5 °C climate of this century
Source: Sci Data. 2025 Dec 11;13:96. doi: 10.1038/s41597-025-06411-9 (PMC12830754; doi:10.1038/s41597-025-06411-9)
Supplement: Supplementary file 1 — Supplementary Information of 1 km HILDA+ based land cover/use map time series of China under 1.5 °C climate of this century [file 41597_2025_6411_MOESM1_ESM.docx]

**Supplementary Information of**

***1 km HILDA+ based land cover/use map time series of China under 1.5 °C climate of this century***

Yifan Gao^1^, Xian Feng^1^, Changqing Song^1^, Yuanhui Wang^1^, Sijing Ye^1^, Min Zhao^1^, Delin Fang^1^, Peichao Gao^1,*^

1. State Key Laboratory of Earth Surface Processes and Disaster Risk Reduction, Faculty of Geographical Science, Beijing Normal University, Beijing 100875, China

*corresponding author(s): Peichao Gao (gaopc@bnu.edu.cn)

**Content**

[Text 5](#_Toc214984742)

[Text1 Selection of the 1.5 °C climate scenario. 5](#_Toc214984743)

[Text2 Land areas changes under the 1.5 °C climate scenario. 6](#_Toc214984744)

[Text3 Comparison of land cover/use changes between the 1.5 °C climate scenario and the no-policy scenario. 8](#_Toc214984745)

[Text4 Impact of historical simulations on future forecasts. 9](#_Toc214984746)

[Figures 10](#_Toc214984747)

[Figure S1 Area changes in five land cover/use types from 2015 to 2100 under the 1.5 °C climate scenario. 10](#_Toc214984748)

[Figure S2 Number of land cover/use type changes for each cell from 2015 to 2100 under 1.5°C climate scenario. 11](#_Toc214984749)

[Figure S3 Summary of cell-by-cell comparison between the 1.5°C climate scenario and no-policy scenario from 2020 to 2100 for the selected ten water basins. 12](#_Toc214984750)

[Figure S4 Cell-by-cell comparison of land cover/use maps between the 1.5°C climate scenario and no-policy scenario in 2050 and 2100 for the selected ten water basins. 13](#_Toc214984751)

[Tables 14](#_Toc214984752)

[Table S1 Dataset materials. 14](#_Toc214984753)

[Table S2 Average and maximum differences between demands and supplies. 16](#_Toc214984754)

[Table S3 AUC of 24 water basins for each land cover/use type. 17](#_Toc214984755)

[Table S4 Neighborhood weight of 24 water basins for each land cover/use type. 19](#_Toc214984756)

[Table S5 Land transformation matrix from 2015 to 2020 under 1.5°C climate scenario (Unit: km^2^). 21](#_Toc214984757)

[Table S6 Land transformation matrix from 2020 to 2030 under 1.5°C climate scenario (Unit: km^2^). 22](#_Toc214984758)

[Table S7 Land transformation matrix from 2030 to 2040 under 1.5°C climate scenario (Unit: km^2^). 23](#_Toc214984759)

[Table S8 Land transformation matrix from 2040 to 2050 under 1.5°C climate scenario (Unit: km^2^). 24](#_Toc214984760)

[Table S9 Land transformation matrix from 2050 to 2060 under 1.5°C climate scenario (Unit: km^2^). 25](#_Toc214984761)

[Table S10 Land transformation matrix from 2060 to 2070 under 1.5°C climate scenario (Unit: km^2^). 26](#_Toc214984762)

[Table S11 Land transformation matrix from 2070 to 2080 under 1.5°C climate scenario (Unit: km^2^). 27](#_Toc214984763)

[Table S12 Land transformation matrix from 2080 to 2090 under 1.5°C climate scenario (Unit: km^2^). 28](#_Toc214984764)

[Table S13 Land transformation matrix from 2090 to 2100 under 1.5°C climate scenario (Unit: km^2^). 29](#_Toc214984765)

[Table S14 Difference in areas and proportions between the 1.5°C climate scenario and the no-policy scenario. 30](#_Toc214984766)

[References 31](#_Toc214984767)

# Text

## Text1 Selection of the 1.5 °C climate scenario.

In this study, we selected the 1.5 °C climate scenario with NDCs. Compared with the scenarios based on SSP or RCP, the scenario with NDCs is more realistic, as NDCs are officially submitted by the parties themselves. Consequently, forecasts with NDCs are able to better reflect consequences by considering the intentions of climate actions. In contrast, scenarios based on SSP and RCP only describe alternative evolutions of future society under different development pathways, forcing levels, and greenhouse gas.

Regarding the scenario selected in this study, we acknowledge that achieving the 1.5 °C target faces challenges, but it still holds importance. First, the 1.5°C target remains central to the international climate policy framework, notably the Paris Agreement. In addition, the United Nations has launched the Climate Promise 2025 initiative to support countries in aligning their NDCs with the 1.5°C target. Second, although the 1.5°C climate scenario entails strong assumptions (all parties would adhere to their submitted NDCs, the decarbonization rate in the 1.5 °C climate scenario is 8% after 2030, and the timeline for achieving net-zero emissions is on schedule), these do not affect our examination of the spatially explicit consequences, which can provide valuable insights for parties seeking to refine or adjust their NDCs. NDCs themselves are dynamic rather than static commitments. Many parties continuously revise or enhance their NDCs over time. Although some parties have withdrawn from the Paris Agreement, the Paris Agreement itself has not failed. On the contrary, the ratcheting-up mechanism to increase ambition is still active. Recent developments, such as the negotiations for a Global Plastics Treaty (https://www.globalplasticaction.org/globalplasticstreaty) and initiatives like the EAT-Lancet diet (https://eatforum.org/eat-lancet-commission/the-planetary-health-diet-and-you/), further highlight the persistence of collective global efforts toward climate mitigation and sustainability.

## Text2 Land areas changes under the 1.5 °C climate scenario.

The potential area changes in land cover/use types under the 1.5 °C climate scenario are shown in Fig. S1. Our results indicate that the cropland will be under substantial pressure of rapid decline after 2030. And compared with 2015, 29.69% of cropland in 2100 is forecasted to be under pressure of decline under the 1.5 °C climate scenario. The pasture/rangeland area in 2100 is forecasted to be under a 6.62% decline compared with the area in 2015. However, this decline pressure is not continuous over time, as the area does not consistently decline before 2100. From 2050 to 2060, the area of pasture/rangeland shows a 0.37% expansion potential. The forest area exhibits a 30.13% expansion potential from 2015 to 2100. The main sources of forest expansion are cropland and pasture/rangeland. In the periods from 2020 to 2050 and from 2060 to 2070, pasture/rangeland was the largest source of forest expansion. Specifically, pasture/rangeland contributes 70.30% (25,032 km^2^) from 2020 to 2030, 60.27% (109,749 km^2^) from 2030 to 2040, 63.20% (115,028 km^2^) from 2040 to 2050, and 53.97% (48,071 km^2^) from 2060 to 2070. During the periods from 2050 to 2060 and from 2070 to 2100, cropland was the largest source of forest expansion. Specifically, cropland contributes 91.38% (65,874 km^2^) from 2050 to 2060, 78.77% (31,729 km^2^) from 2070 to 2080, 80.45% (34,034 km^2^) from 2080 to 2090, and 95.24% (19,486 km^2^) from 2090 to 2100. The areas of unmanaged grass/shrubland and sparse/no vegetation increased by 1.12% and 1.19%, respectively.

This study focuses on the potential spatially explicit consequences of land cover/use under the 1.5 °C climate scenario with taking into account NDCs, without taking into account certain national policies (e.g., China’s cropland red line). Nevertheless, our results indicate that such national policies play a critical role. Specifically, Fig. S1 illustrates the substantial pressure on cropland decline. In the absence of China’s cropland red line, cropland would be at considerable risk. To prevent the decline of cropland, it is necessary to implement protection policies such as China’s cropland red line. China’s cropland red line serves as a crucial cornerstone for ensuring the stability of cropland and safeguarding food security. Furthermore, our results underscore that government decision-making should account for both international commitments and domestic development needs.

According to our forecasts of land cover/use maps, more obvious land cover/use changes in the future will occur from 2030 to 2060 (Tables S5-13). From 2030 to 2040, land cover/use changes cover an area of 201,568 km^2^, accounting for 25.1% of the total land cover/use changes. Specifically, 5.1% (109,749 km^2^) of the pasture/rangeland area will be changed to forest. A total of 2.8% (69,147 km^2^) of cropland will be changed to forest. In addition, 0.6% (8,715 km^2^) of sparse/no vegetation will be changed to pasture/rangeland. From 2040 to 2050, land cover/use changes will cover an area of 194,326 km^2^, accounting for 24.2% of the total land cover/use changes. Specifically, 5.1% (64,692 km^2^) of the cropland and 3.0% (115,028 km^2^) of pasture/rangeland will be changed to forest. From 2050 to 2060, land cover/use changes will cover an area of 109,064 km^2^, accounting for 13.6% of total land cover/use changes. In particular, a total of 96,758 km² of cropland will be changed to other land cover/use types, marking the fastest period of cropland loss. Specifically, 5.4% (65,874 km^2^) of cropland will be changed to forest. A total of 1.0% (12,547 km^2^) of cropland will be changed to sparse/no vegetation. A total of 1.5% (18,234 km^2^) of cropland will be changed to pasture/rangeland.

Our forecasts of land cover/use maps reveal notable heterogeneity in both the areas of change and the spatial patterns of land cover/use changes forecasted to have occurred before 2100. According to our forecasts of land cover/use maps, an area of 784,808 km^2^ (8.3%) in China is forecasted to experience at least one change before 2100. Among the regions that experience at least one change, 97.8% (767,386 km^2^) of areas experience only one change before 2100. In addition, 2.2% (16,899 km^2^) of the areas experienced two changes before 2100. Only 0.1% (523 km^2^) of areas experience three changes before 2100. In terms of spatial distribution, regions experiencing one land cover/use type change are located mainly in the northern, northeastern, and southern regions of China (Fig. S1). Regions experiencing two or three changes are predominantly concentrated in the Bo Hai Korean Bay North Coast (Fig. S2).

## Text3 Comparison of land cover/use changes between the 1.5 °C climate scenario and the no-policy scenario.

To clarify how the 1.5 °C climate scenario modifies land cover/use patterns relative to the baseline, we selected a no-policy scenario^1^, which assumes no new emission mitigation policies through 2100. This scenario indicates approximately a 30% likelihood of a 2‒3 °C increase in global temperature relative to the pre-industrial level, a 54% likelihood of a 3‒4 °C increase, and a 16% likelihood of exceeding 4 °C. Using the no-policy scenario, we first calculated future demands. We then selected 10 water basins that exhibited the largest demand differences compared to the 1.5 °C climate scenario**.** These basins consist of the Ob, Lake Balkash, Ziya He Interior, China Coast, Indus, Taiwan, Yangtze, Xun Jiang, Hong (Red River), and South China Sea Coast. Subsequently, for these selected water basins, we simulated spatially explicit land cover/use changes at a ten-year interval.

Comparison of the 1.5 °C climate scenario with the no-policy scenario reveals a divergence exceeding 4% after 2040 (Table S14). The differences are mainly reflected in cropland, forest, and pasture/rangeland (Figs. S3-4). In 2040, under the no-policy scenario, 9.66% of the areas forecasted as cropland and 4.69% of the areas forecasted as pasture/rangeland are forecasted as forest in the 1.5 °C climate scenario. Both the proportions exhibit an increasing trend after 2040. And in 2100, under the no-policy scenario, 17.24% of the areas forecasted as cropland and 8.78% of the areas forecasted as pasture/rangeland are forecasted as forest in the 1.5 °C climate scenario.

## Text4 Impact of historical simulations on future forecasts.

Regarding the uncertainty in future forecasts, it is difficult to evaluate the impact of historical simulations, as both FoM and Kappa vary when different historical periods are used for evaluations. Therefore, historical simulation performance may not directly impact the uncertainty of future forecasts.

# Figures

## Figure S1 Area changes in five land cover/use types from 2015 to 2100 under the 1.5 °C climate scenario. These land areas were derived from the statistical analysis of the forecasts of land cover/use maps in China. Because urban and water areas are assumed to remain unchanged in the simulation, only the area changes in the other five land cover/use types are illustrated. a. Changes in the areas of cropland. b. Changes in the areas of pasture/rangeland. c. Changes in the area of forest. d. Changes in the area of unmanaged grass/shrubland. e. Changes in the area of sparse/no vegetation. Land areas for each water basin are shown in the supplementary materials.


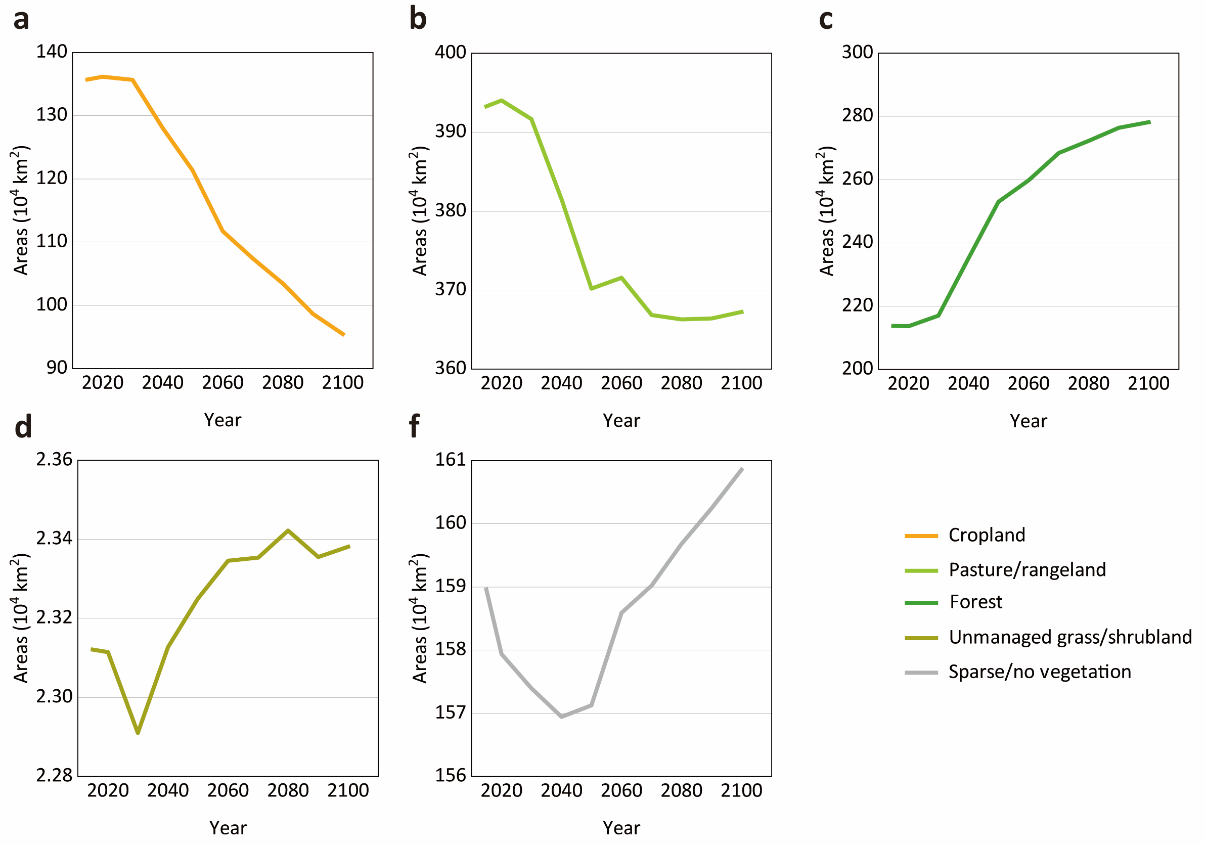


## Figure S2 Number of land cover/use type changes for each cell from 2015 to 2100 under 1.5°C climate scenario. Blank regions indicate the cells without land cover/use type changes. The China boundary can be accessed at <https://cloudcenter.tianditu.gov.cn/administrativeDivision/>.


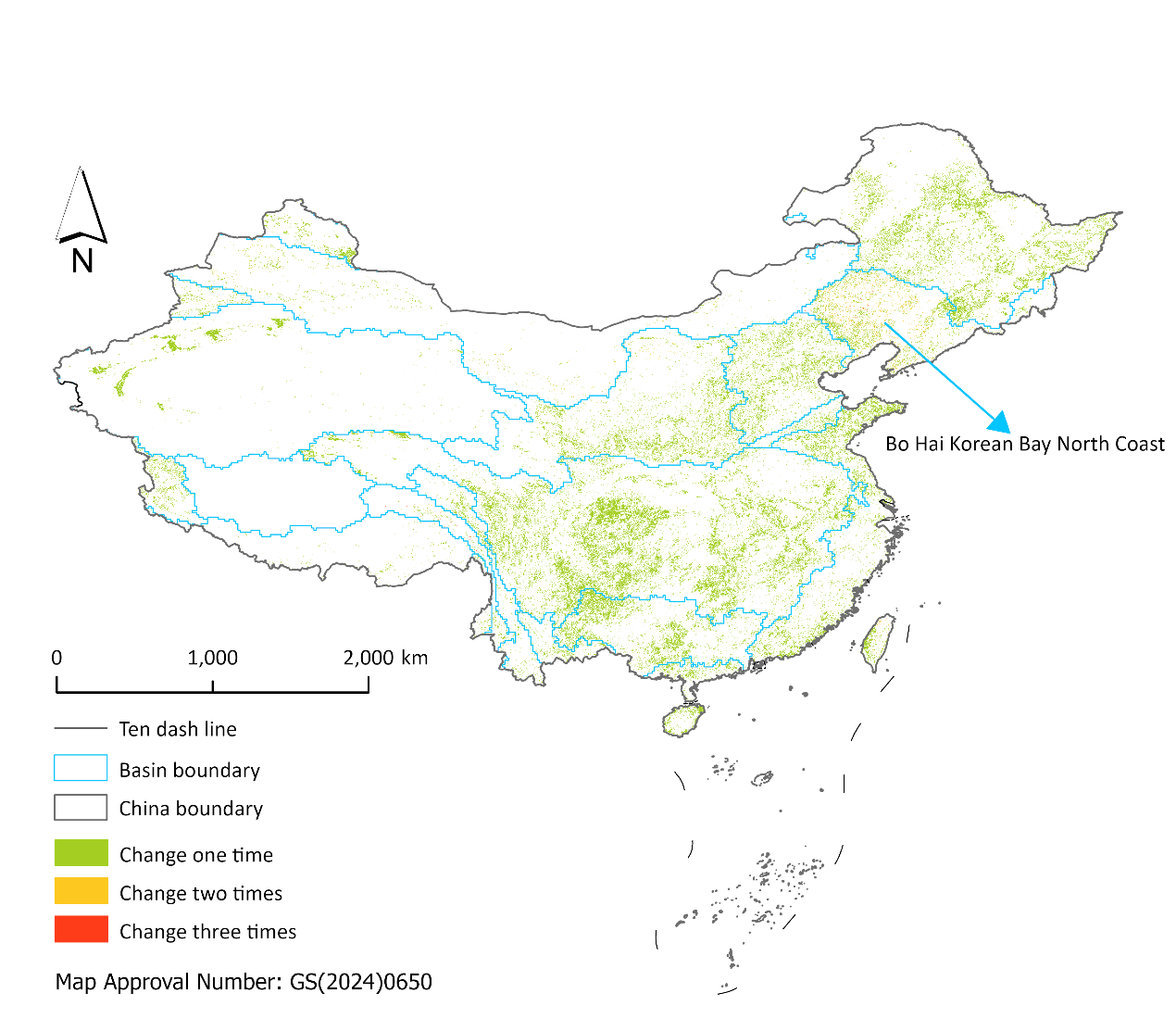


## Figure S3 Summary of cell-by-cell comparison between the 1.5°C climate scenario and no-policy scenario from 2020 to 2100 for the selected ten water basins.


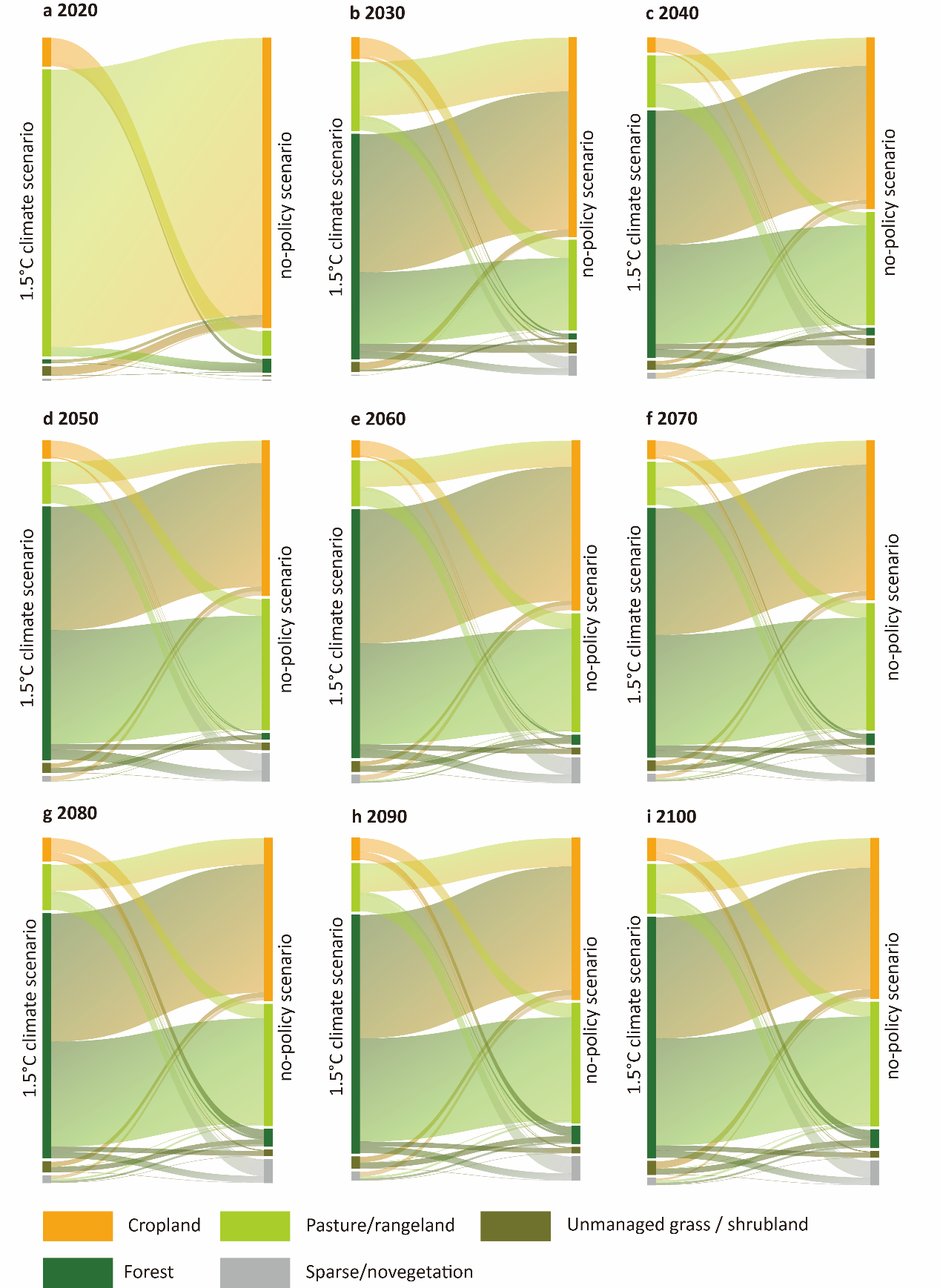


## Figure S4 Cell-by-cell comparison of land cover/use maps between the 1.5°C climate scenario and no-policy scenario in 2050 and 2100 for the selected ten water basins. a. Land change map in 2050 under the 1.5°C climate scenario. b. Land change map in 2050 under the no-policy scenario. c. Land change map in 2100 under the 1.5°C climate scenario. d. Land change map in 2100 under the no-policy scenario. Colorful areas indicate cells where land cover/use type differs between the two scenarios, while blank areas indicate cells where land cover/use type remains the same.


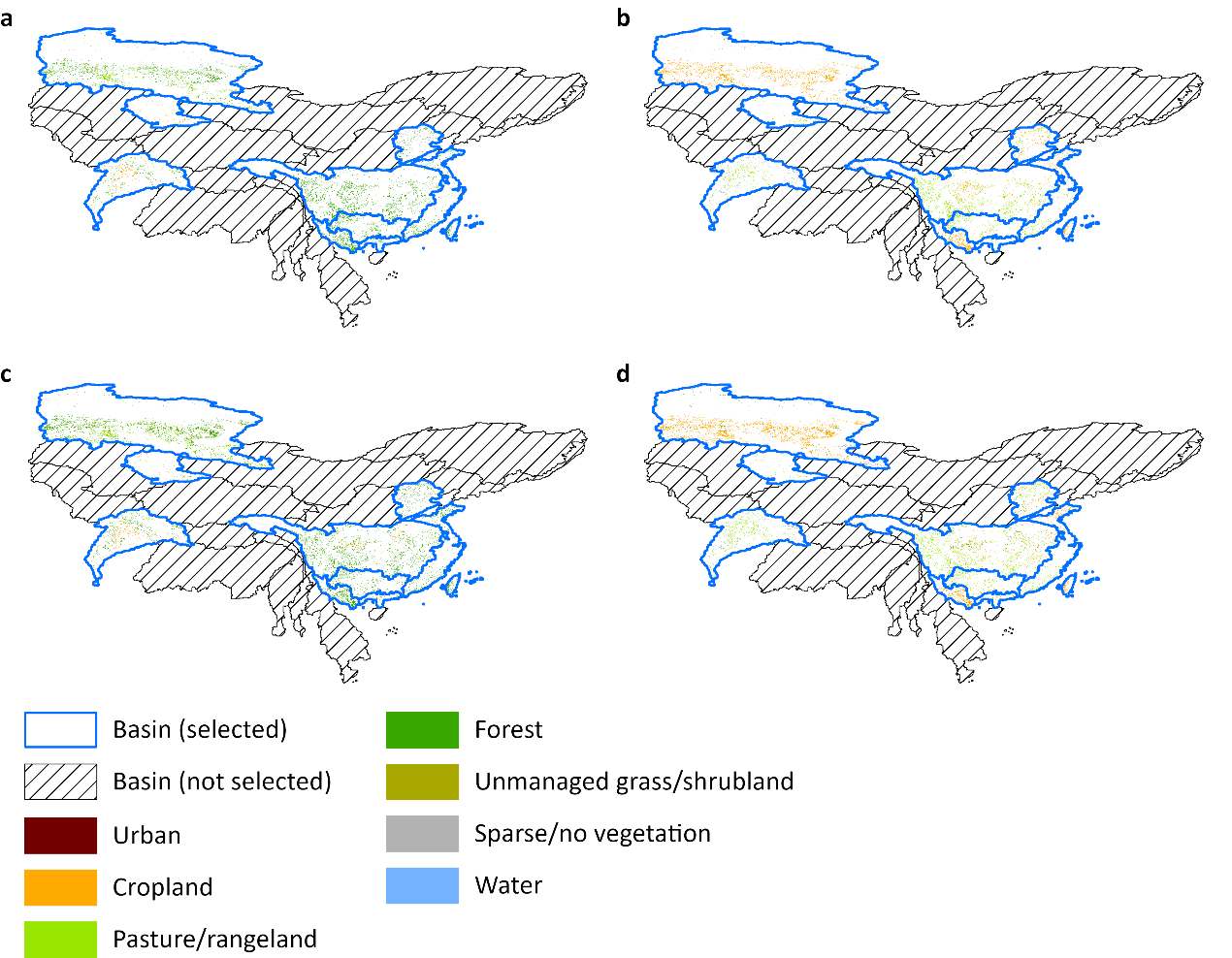


# Tables

## Table S1 Dataset materials.

| Category | | Data | Source |
| --- | --- | --- | --- |
| Land cover/use map | | HIstoric Land Dynamics Assessment+ | Winkler, et al. ^2,3^ |
| Driving factors | Soil | Bulk density | Hengl, et al. ^4^ |
|  |  | Cation exchange capacity |  |
|  |  | Clay content |  |
|  |  | Coarse fragments volumetric |  |
|  |  | Derived available soil water capacity |  |
|  |  | Organic carbon density |  |
|  |  | PH in H_2_O |  |
|  |  | Sand content |  |
|  |  | Silt content |  |
|  |  | Texture class |  |
|  | Socio-economic | Market access index | Verburg, et al. ^5^ |
|  |  | Market influence index ($/person) |  |
|  |  | Market density index |  |
|  |  | Nighttime lights | DMSP-OLS Nighttime Lights Time Series Version 4 (https://datadryad.org/stash/dataset/doi:10.5061/dryad.dk1j0) |
|  |  | Total GDP (PPP, purchasing power parity) | Kummu, et al. ^6^ |
|  |  | Gridded Population of the World (GPW) | NASA Socioeconomic Data and Applications Center (SEDAC) () |
|  | Accessibility | Time to nearest cities | Weiss, et al. ^7^ |
|  |  | Rivers and lake centerlines | Natural Earth (https://www.naturalearthdata.com/) |
|  |  | Distance to the nearest railway |  |
|  |  | Railroads |  |
|  |  | Travel time one meter (motorized) | Weiss, et al. ^8^ |
|  |  | Travel time one meter (walking-only) |  |
|  |  | Time to nearest healthcare facility (motorized) |  |
|  |  | Time to nearest healthcare facility (walking-only) |  |
|  | Agriculture and Vegetation | 175 Crops Yield per hectare | Monfreda, et al. ^9^ |
|  |  | Gross primary production-March | Wang and Zhang ^10^ |
|  |  | Gross primary production-June |  |
|  |  | Gross primary production-September |  |
|  |  | Gross primary production-December |  |
|  |  | NDVI-March | NASA EOSDIS Land Processes DAAC (https://land.copernicus.eu/global/) |
|  |  | NDVI- June |  |
|  |  | NDVI- September |  |
|  |  | NDVI- December |  |
|  | Terrain | Elevation | Fick and Hijmans ^11^ |
|  |  | variance of elevation | Calculated by elevation |
|  |  | Slope |  |
|  |  | Aspect |  |
|  | Climate | Annual mean precipitation | Hengl ^12^ |
|  |  | Mean precipitation-March |  |
|  |  | Mean precipitation-June |  |
|  |  | Mean precipitation-September |  |
|  |  | Mean precipitation-December |  |
|  |  | Annual mean temperature | Hengl ^13^ |
|  |  | Mean temperature-March |  |
|  |  | Mean temperature-June |  |
|  |  | Mean temperature-September |  |
|  |  | Mean temperature-December |  |
|  | Livestock | Buffaloes | Gridded Livestock of the World – 2010 (GLW 3) (https://dataverse.harvard.edu/dataverse/glw) |
|  |  | Cattle |  |
|  |  | Chickens |  |
|  |  | Ducks |  |
|  |  | Goats |  |
|  |  | Horses |  |
|  |  | Pigs |  |
|  |  | Sheep |  |

## Table S2 Average and maximum differences between demands and supplies.

| Water basin | Maximum difference | Average difference |
| --- | --- | --- |
| Russia South East Coast | 1.06 | 1.01 |
| Gobi Interior | 0.45 | 0.24 |
| Amur | 0.31 | 0.11 |
| Ob | 0.26 | 0.13 |
| Amu Darya | 0.50 | 0.16 |
| Bo Hai Korean Bay North Coast | 0.50 | 0.20 |
| Lake Balkash | 0.66 | 0.29 |
| Syr Darya | 0.36 | 0.23 |
| Ziya He Interior | 0.32 | 0.30 |
| China Coast | 0.54 | 0.46 |
| Huang He | 0.36 | 0.25 |
| Tarim Interior | 0.51 | 0.24 |
| Indus | 0.54 | 0.22 |
| Plateau of Tibet Interior | 0.07 | 0.07 |
| Taiwan | 0.43 | 0.29 |
| Yangtze | 0.56 | 0.50 |
| Xun Jiang | 0.93 | 0.80 |
| Hong (Red River) | 0.12 | 0.07 |
| Ganges Bramaputra | 0.48 | 0.25 |
| South China Sea Coast | 1.34 | 1.31 |
| Hainan | 0.76 | 0.39 |
| Mekong | 0.55 | 0.26 |
| Salween | 0.49 | 0.17 |
| Irrawaddy | 0.38 | 0.25 |

## Table S3 AUC of 24 water basins for each land cover/use type. “-” indicates that there are insufficient samples to implement regressions.

| Water basins | Urban | Cropland | Pasture/rangeland | Forest | Unmanaged grass/shrubland | Sparse/no vegetation | Water |
| --- | --- | --- | --- | --- | --- | --- | --- |
| Russia South East Coast | 0.69 | 0.85 | 0.60 | 0.88 | 0.88 | 1.00 | - |
| Gobi Interior | 0.93 | 0.97 | 0.97 | 0.98 | 0.88 | 0.94 | - |
| Amur | 0.76 | 0.87 | 0.91 | 0.95 | 0.95 | 0.62 | - |
| Ob | 0.71 | 0.92 | 0.92 | 0.86 | 0.91 | 0.84 | - |
| Amu Darya | 0.92 | 0.89 | 0.92 | 0.96 | 0.91 | 0.92 | - |
| Bo Hai Korean Bay North Coast | 0.82 | 0.88 | 0.72 | 0.95 | 0.94 | - | - |
| Lake Balkash | 0.85 | 0.92 | 0.97 | 0.97 | 0.82 | 0.95 | - |
| Syr Darya | 0.88 | 0.95 | 0.94 | 0.95 | 0.83 | 0.95 | - |
| Ziya He Interior | 0.93 | 0.96 | 0.73 | 0.95 | - | - | - |
| China Coast | 0.88 | 0.80 | 0.76 | 0.84 | 0.75 | - | - |
| Huang He | 0.93 | 0.96 | 0.92 | 0.96 | - | 0.50 | - |
| Tarim Interior | 0.95 | 0.98 | 0.91 | 0.98 | 0.50 | 0.77 | - |
| Indus | 0.89 | 0.95 | 0.92 | 0.97 | 0.93 | 0.95 | - |
| Plateau of Tibet Interior | 1.00 | - | 0.85 | - | - | 0.72 | - |
| Taiwan | - | - | - | 0.75 | - | - | - |
| Yangtze | 0.80 | 0.80 | 0.85 | 0.91 | - | 0.75 | - |
| Xun Jiang | 0.80 | 0.81 | 0.71 | 0.88 | 0.73 | - | - |
| Hong (Red River) | 0.85 | 0.92 | 0.67 | 0.93 | 0.86 | - | - |
| Ganges Bramaputra | 0.88 | 0.92 | 0.93 | 0.95 | 0.94 | 0.96 | - |
| South China Sea Coast | 0.85 | 0.92 | 0.81 | 0.89 | 0.59 | - | - |
| Hainan | 0.56 | 0.78 | 0.60 | 0.92 | - | - | - |
| Mekong | 0.90 | 0.95 | 0.79 | 0.94 | 0.93 | 0.79 | - |
| Salween | 0.69 | 0.95 | 0.64 | 0.92 | 0.89 | 0.50 | - |
| Irrawaddy | 0.77 | 0.94 | 0.69 | 0.91 | 0.90 | 0.70 | - |

## Table S4 Neighborhood weight of 24 water basins for each land cover/use type.

| Water basins | Urban | Cropland | Pasture/rangeland | Forest | Unmanaged grass/shrubland | Sparse/no vegetation | Water |
| --- | --- | --- | --- | --- | --- | --- | --- |
| Russia South East Coast | 1 | 1 | 1 | 1 | 1 | 1 | 1 |
| Gobi Interior | 1 | 1 | 1 | 1 | 1 | 1 | 1 |
| Amur | 1 | 1 | 1 | 1 | 1 | 1 | 1 |
| Ob | 1 | 1 | 1 | 1 | 1 | 1 | 1 |
| Amu Darya | 1 | 1 | 1 | 1 | 1 | 1 | 1 |
| Bo Hai Korean Bay North Coast | 1 | 1 | 1 | 1 | 1 | 1 | 1 |
| Lake Balkash | 1 | 1 | 1 | 1 | 1 | 1 | 1 |
| Syr Darya | 0 | 0 | 0 | 0 | 0 | 0 | 0 |
| Ziya He Interior | 1 | 1 | 1 | 1 | 1 | 1 | 1 |
| China Coast | 1 | 1 | 1 | 1 | 1 | 1 | 1 |
| Huang He | 1 | 1 | 1 | 1 | 1 | 1 | 1 |
| Tarim Interior | 0 | 0 | 0 | 0 | 0 | 0 | 0 |
| Indus | 1 | 1 | 1 | 1 | 1 | 1 | 1 |
| Plateau of Tibet Interior | 0 | 0 | 0 | 0 | 0 | 0 | 0 |
| Taiwan | 1 | 1 | 1 | 1 | 1 | 1 | 1 |
| Yangtze | 1 | 1 | 1 | 1 | 1 | 1 | 1 |
| Xun Jiang | 1 | 1 | 1 | 1 | 1 | 1 | 1 |
| Hong (Red River) | 0 | 0 | 0 | 0 | 0 | 0 | 0 |
| Ganges Bramaputra | 1 | 1 | 1 | 1 | 1 | 1 | 1 |
| South China Sea Coast | 1 | 1 | 1 | 1 | 1 | 1 | 1 |
| Hainan | 1 | 1 | 1 | 1 | 1 | 1 | 1 |
| Mekong | 0 | 0 | 0 | 0 | 0 | 0 | 0 |
| Salween | 1 | 1 | 1 | 1 | 1 | 1 | 1 |
| Irrawaddy | 1 | 1 | 1 | 1 | 1 | 1 | 1 |

## Table S5 Land transformation matrix from 2015 to 2020 under 1.5°C climate scenario (Unit: km^2^).

| Year | 2020 | | | | | | | |
| --- | --- | --- | --- | --- | --- | --- | --- | --- |
| 2015 | Land cover/use type | Urban | Cropland | Pasture/rangeland | Forest | Unmanaged grass/shrubland | Sparse/no vegetation | Water |
|  | Urban | 339,659 | 0 | 0 | 0 | 0 | 0 | 0 |
|  | Cropland | 0 | 1,357,398 | 2 | 23 | 0 | 0 | 0 |
|  | Pasture/rangeland | 0 | 1,380 | 3,931,253 | 38 | 0 | 3 | 0 |
|  | Forest | 0 | 699 | 677 | 2,136,364 | 0 | 0 | 0 |
|  | Unmanaged grass/shrubland | 0 | 6 | 0 | 0 | 23,115 | 0 | 0 |
|  | Sparse/no vegetation | 0 | 2,075 | 8,147 | 0 | 0 | 15,793,51 | 0 |
|  | Water | 0 | 0 | 0 | 0 | 0 | 0 | 109,595 |
| Total change: 13,050 | | | | | | | | |

## Table S6 Land transformation matrix from 2020 to 2030 under 1.5°C climate scenario (Unit: km^2^).

| Year | 2030 | | | | | | | |
| --- | --- | --- | --- | --- | --- | --- | --- | --- |
| 2020 | Land cover/use type | Urban | Cropland | Pasture/rangeland | Forest | Unmanaged grass/shrubland | Sparse/no vegetation | Water |
|  | Urban | 339,659 | 0 | 0 | 0 | 0 | 0 | 0 |
|  | Cropland | 0 | 1,352,231 | 42 | 9,285 | 0 | 0 | 0 |
|  | Pasture/rangeland | 0 | 2,421 | 3,912,611 | 25,032 | 0 | 15 | 0 |
|  | Forest | 0 | 1,627 | 0 | 2,134,798 | 0 | 0 | 0 |
|  | Unmanaged grass/shrubland | 0 | 12 | 0 | 193 | 22,910 | 0 | 0 |
|  | Sparse/no vegetation | 0 | 535 | 3,784 | 1,099 | 0 | 1,573,936 | 0 |
|  | Water | 0 | 0 | 0 | 0 | 0 | 0 | 109,595 |
| Total change: 44,045 | | | | | | | | |

## Table S7 Land transformation matrix from 2030 to 2040 under 1.5°C climate scenario (Unit: km^2^).

| Year | 2040 | | | | | | | |
| --- | --- | --- | --- | --- | --- | --- | --- | --- |
| 2030 | Land cover/use type | Urban | Cropland | Pasture/rangeland | Forest | Unmanaged grass/shrubland | Sparse/no vegetation | Water |
|  | Urban | 339,659 | 0 | 0 | 0 | 0 | 0 | 0 |
|  | Cropland | 0 | 1,279,131 | 1,227 | 69,147 | 0 | 7,321 | 0 |
|  | Pasture/rangeland | 0 | 1,227 | 3,805,302 | 109,749 | 0 | 159 | 0 |
|  | Forest | 0 | 0 | 0 | 2,169,983 | 424 | 0 | 0 |
|  | Unmanaged grass/shrubland | 0 | 1 | 14 | 190 | 22,657 | 48 | 0 |
|  | Sparse/no vegetation | 0 | 335 | 8715 | 2,965 | 46 | 1,561,890 | 0 |
|  | Water | 0 | 0 | 0 | 0 | 0 | 0 | 109,595 |
| Total change: 201,568 | | | | | | | | |

## Table S8 Land transformation matrix from 2040 to 2050 under 1.5°C climate scenario (Unit: km^2^).

| Year | 2050 | | | | | | | |
| --- | --- | --- | --- | --- | --- | --- | --- | --- |
| 2040 | Land cover/use type | Urban | Cropland | Pasture/rangeland | Forest | Unmanaged grass/shrubland | Sparse/no vegetation | Water |
|  | Urban | 339,659 | 0 | 0 | 0 | 0 | 0 | 0 |
|  | Cropland | 0 | 1,211,469 | 76 | 64,692 | 0 | 4,457 | 0 |
|  | Pasture/rangeland | 0 | 447 | 3,699,777 | 115,028 | 0 | 6 | 0 |
|  | Forest | 0 | 0 | 0 | 2,348,462 | 345 | 3,227 | 0 |
|  | Unmanaged grass/shrubland | 0 | 0 | 1 | 220 | 22,905 | 1 | 0 |
|  | Sparse/no vegetation | 0 | 1,834 | 1,935 | 2,057 | 0 | 1,563,592 | 0 |
|  | Water | 0 | 0 | 0 | 0 | 0 | 0 | 109,595 |
| Total change: 194,326 | | | | | | | | |

## Table S9 Land transformation matrix from 2050 to 2060 under 1.5°C climate scenario (Unit: km^2^).

| Year | 2060 | | | | | | | |
| --- | --- | --- | --- | --- | --- | --- | --- | --- |
| 2050 | Land cover/use type | Urban | Cropland | Pasture/rangeland | Forest | Unmanaged grass/shrubland | Sparse/no vegetation | Water |
|  | Urban | 339,659 | 0 | 0 | 0 | 0 | 0 | 0 |
|  | Cropland | 0 | 1,116,992 | 18,234 | 65,874 | 103 | 12,547 | 0 |
|  | Pasture/rangeland | 0 | 91 | 3,695,113 | 5,996 | 2 | 587 | 0 |
|  | Forest | 0 | 373 | 1,056 | 2,526,199 | 0 | 2,831 | 0 |
|  | Unmanaged grass/shrubland | 0 | 0 | 1 | 0 | 23,241 | 8 | 0 |
|  | Sparse/no vegetation | 0 | 27 | 1,114 | 220 | 0 | 1,569,922 | 0 |
|  | Water | 0 | 0 | 0 | 0 | 0 | 0 | 109,595 |
| Total change: 109,064 | | | | | | | | |

## Table S10 Land transformation matrix from 2060 to 2070 under 1.5°C climate scenario (Unit: km^2^).

| Year | 2070 | | | | | | | |
| --- | --- | --- | --- | --- | --- | --- | --- | --- |
| 2060 | Land cover/use type | Urban | Cropland | Pasture/rangeland | Forest | Unmanaged grass/shrubland | Sparse/no vegetation | Water |
|  | Urban | 339,659 | 0 | 0 | 0 | 0 | 0 | 0 |
|  | Cropland | 0 | 1,073,592 | 301 | 40,943 | 0 | 2,647 | 0 |
|  | Pasture/rangeland | 0 | 0 | 3,667,248 | 48,071 | 11 | 188 | 0 |
|  | Forest | 0 | 233 | 314 | 2,595,471 | 6 | 2,265 | 0 |
|  | Unmanaged grass/shrubland | 0 | 0 | 0 | 7 | 23,337 | 2 | 0 |
|  | Sparse/no vegetation | 0 | 5 | 696 | 54 | 0 | 1,585,140 | 0 |
|  | Water | 0 | 0 | 0 | 0 | 0 | 0 | 109,595 |
| Total change: 95,743 | | | | | | | | |

## Table S11 Land transformation matrix from 2070 to 2080 under 1.5°C climate scenario (Unit: km^2^).

| Year | 2080 | | | | | | | |
| --- | --- | --- | --- | --- | --- | --- | --- | --- |
| 2070 | Land cover/use type | Urban | Cropland | Pasture/rangeland | Forest | Unmanaged grass/shrubland | Sparse/no vegetation | Water |
|  | Urban | 339,659 | 0 | 0 | 0 | 0 | 0 | 0 |
|  | Cropland | 0 | 1,034,591 | 1,790 | 31,729 | 30 | 5,690 | 0 |
|  | Pasture/rangeland | 0 | 0 | 3,660,122 | 8,434 | 0 | 3 | 0 |
|  | Forest | 0 | 0 | 182 | 2,682,709 | 0 | 1,655 | 0 |
|  | Unmanaged grass/shrubland | 0 | 0 | 0 | 0 | 23,354 | 0 | 0 |
|  | Sparse/no vegetation | 0 | 0 | 670 | 63 | 38 | 1,589,471 | 0 |
|  | Water | 0 | 0 | 0 | 0 | 0 | 0 | 109,595 |
| Total change: 50,284 | | | | | | | | |

## Table S12 Land transformation matrix from 2080 to 2090 under 1.5°C climate scenario (Unit: km^2^).

| Year | 2090 | | | | | | | |
| --- | --- | --- | --- | --- | --- | --- | --- | --- |
| 2080 | Land cover/use type | Urban | Cropland | Pasture/rangeland | Forest | Unmanaged grass/shrubland | Sparse/no vegetation | Water |
|  | Urban | 339,659 | 0 | 0 | 0 | 0 | 0 | 0 |
|  | Cropland | 0 | 986,384 | 7,870 | 34,034 | 20 | 6,283 | 0 |
|  | Pasture/rangeland | 0 | 0 | 3,655,005 | 7,759 | 0 | 0 | 0 |
|  | Forest | 0 | 0 | 35 | 2,721,839 | 1 | 1,060 | 0 |
|  | Unmanaged grass/shrubland | 0 | 0 | 1 | 87 | 23,334 | 0 | 0 |
|  | Sparse/no vegetation | 0 | 0 | 1,243 | 422 | 0 | 1,595,154 | 0 |
|  | Water | 0 | 0 | 0 | 0 | 0 | 0 | 109,595 |
| Total change: 58,815 | | | | | | | | |

## Table S13 Land transformation matrix from 2090 to 2100 under 1.5°C climate scenario (Unit: km^2^).

| Year | 2100 | | | | | | | |
| --- | --- | --- | --- | --- | --- | --- | --- | --- |
| 2090 | Land cover/use type | Urban | Cropland | Pasture/rangeland | Forest | Unmanaged grass/shrubland | Sparse/no vegetation | Water |
|  | Urban | 339,659 | 0 | 0 | 0 | 0 | 0 | 0 |
|  | Cropland | 0 | 954,420 | 8,802 | 19,486 | 29 | 3,647 | 0 |
|  | Pasture/rangeland | 0 | 0 | 3,663,197 | 957 | 0 | 0 | 0 |
|  | Forest | 0 | 0 | 129 | 2,761,398 | 3 | 2,611 | 0 |
|  | Unmanaged grass/shrubland | 0 | 0 | 1 | 5 | 23,349 | 0 | 0 |
|  | Sparse/no vegetation | 0 | 0 | 176 | 12 | 0 | 1,602,309 | 0 |
|  | Water | 0 | 0 | 0 | 0 | 0 | 0 | 109,595 |
| Total change: 35,858 | | | | | | | | |

## Table S14 Difference in areas and proportions between the 1.5°C climate scenario and the no-policy scenario.

| Year | Differences in area (km^2^) | Proportion (%) |
| --- | --- | --- |
| 2020 | 9,122 | 0.12 |
| 2030 | 97,627 | 1.25 |
| 2040 | 329,617 | 4.21 |
| 2050 | 490,329 | 6.26 |
| 2060 | 521,711 | 6.66 |
| 2070 | 565,257 | 7.22 |
| 2080 | 573,426 | 7.32 |
| 2090 | 574,278 | 7.33 |
| 2100 | 574,006 | 7.33 |

# References

1 Ou, Y., Iyer, G., Clarke, L., Edmonds, J., Fawcett, A. A., Hultman, N., McFarland, J. R., Binsted, M., Cui, R., Fyson, C. Can updated climate pledges limit warming well below 2° C? *Science* **374**, 693-695, (2021).

2 Winkler, K., Fuchs, R., Rounsevell, M., Herold, M. Global land use changes are four times greater than previously estimated. *Nature communications* **12**, 2501, (2021).

3 Winkler, K., Fuchs, R., Rounsevell, M. D. A., Herold, M. HILDA+ Global Land Use Change between 1960 and 2019. *PANGAEA* doi:<https://doi.org/10.1594/PANGAEA.921846> (2020).

4 Hengl, T., Mendes de Jesus, J., Heuvelink, G. B., Ruiperez Gonzalez, M., Kilibarda, M., Blagotić, A., Shangguan, W., Wright, M. N., Geng, X., Bauer-Marschallinger, B. SoilGrids250m: Global gridded soil information based on machine learning. *PLoS one* **12**, e0169748, (2017).

5 Verburg, P. H., Ellis, E. C., Letourneau, A. A global assessment of market accessibility and market influence for global environmental change studies. *Environmental Research Letters* **6**, 034019, (2011).

6 Kummu, M., Taka, M., Guillaume, J. H. Gridded global datasets for gross domestic product and Human Development Index over 1990–2015. *Scientific data* **5**, 1-15, (2018).

7 Weiss, D. J., Nelson, A., Gibson, H., Temperley, W., Peedell, S., Lieber, A., Hancher, M., Poyart, E., Belchior, S., Fullman, N. A global map of travel time to cities to assess inequalities in accessibility in 2015. *Nature* **553**, 333-336, (2018).

8 Weiss, D., Nelson, A., Vargas-Ruiz, C., Gligorić, K., Bavadekar, S., Gabrilovich, E., Bertozzi-Villa, A., Rozier, J., Gibson, H., Shekel, T. Global maps of travel time to healthcare facilities. *Nature medicine* **26**, 1835-1838, (2020).

9 Monfreda, C., Ramankutty, N., Foley, J. A. Farming the planet: 2. Geographic distribution of crop areas, yields, physiological types, and net primary production in the year 2000. *Global biogeochemical cycles* **22**, (2008).

10 Wang, S., Zhang, Y. Global gross primary production dataset based on NIRv. *National Tibetan Plateau Data Center* doi:<https://doi.org/10.6084/m9.figshare.12981977.v2> (2020).

11 Fick, S. E., Hijmans, R. J. WorldClim 2: new 1‐km spatial resolution climate surfaces for global land areas. *International journal of climatology* **37**, 4302-4315, (2017).

12 Hengl, T. Monthly precipitation in mm at 1 km resolution based on SM2RAIN-ASCAT 2007-2018, IMERGE, CHELSA Climate and WorldClim (Version 0.2). *Zenodo* doi:<http://doi.org/10.5281/zenodo.3256275> (2018).

13 Hengl, T. Long‐term MODIS LST day‐time and night‐time temperatures, sd and differences at 1 km based on the 2000–2017 time series. (2018).
